# Animal and plant protein intake during infancy and childhood DNA methylation: a meta-analysis in the NutriPROGRAM consortium

**DOI:** 10.1080/15592294.2023.2299045

**Published:** 2024-01-10

**Authors:** Mohammed El Sharkawy, Janine F. Felix, Veit Grote, Trudy Voortman, Vincent W. V. Jaddoe, Berthold Koletzko, Leanne K. Küpers

**Affiliations:** a Division of Metabolic and Nutritional Medicine, Department of Pediatrics, Dr. Von Hauner Children’s Hospital, LMU University Hospital Munich, Munich, Germany; b Munich Medical Research School, Faculty of Medicine, LMU - Ludwig-Maximilians Universität Munich, Munich, Germany; c The Generation R Study Group, Erasmus MC, University Medical Center Rotterdam, Rotterdam, The Netherlands; d Department of Pediatrics, Erasmus MC, University Medical Center Rotterdam, Rotterdam, The Netherlands; e Department of Epidemiology, Erasmus MC, University Medical Center Rotterdam, Rotterdam, The Netherlands

**Keywords:** Epigenetics, childhood DNA methylation, animal protein, early life nutrition, epigenome wide association study

## Abstract

**Background:**

Higher early-life animal protein intake is associated with a higher childhood obesity risk compared to plant protein intake. Differential DNA methylation may represent an underlying mechanism.

**Methods:**

We analysed associations of infant animal and plant protein intakes with DNA methylation in early (2−6 years, *N* = 579) and late (7̄−12 years, *N* = 604) childhood in two studies. Study-specific robust linear regression models adjusted for relevant confounders were run, and then meta-analysed using a fixed-effects model. We also performed sex-stratified meta-analyses. Follow-up analyses included pathway analysis and eQTM look-up.

**Results:**

Infant animal protein intake was not associated with DNA methylation in early childhood, but was associated with late-childhood DNA methylation at cg21300373 (*P* = 4.27 × 10¯^8^, *MARCHF1*) and cg10633363 (*P* = 1.09 × 10¯^7^, *HOXB9*) after FDR correction. Infant plant protein intake was associated with early-childhood DNA methylation at cg25973293 (*P* = 2.26 × 10^−7^, *C1orf159*) and cg15407373 (*P* = 2.13 × 10^−7^, *MBP*) after FDR correction. There was no overlap between the findings from the animal and plant protein analyses. We did not find enriched functional pathways at either time point using CpGs associated with animal and plant protein. These CpGs were not previously associated with childhood gene expression. Sex-stratified meta-analyses showed sex-specific DNA methylation associations for both animal and plant protein intake.

**Conclusion:**

Infant animal protein intake was associated with DNA methylation at two CpGs in late childhood. Infant plant protein intake was associated with DNA methylation in early childhood at two CpGs. A potential mediating role of DNA methylation at these CpGs between infant protein intake and health outcomes requires further investigation.

## Introduction

Dietary composition plays a crucial role in development during the early years of childhood. Protein intake is of particular importance as a source of essential amino acids [1]. Recent observational studies and randomized controlled trials confirmed that high protein intake in early life increases early weight gain and the risk of later overweight and obesity, while growth can be restricted at lower intakes [2,3]. Although the exact pathophysiological mechanisms are unclear, some studies have shown stronger associations of animal protein as compared to plant protein, especially during the first year of life, with higher body mass index (BMI) in later childhood [4,5] and especially higher body fat mass [4–7]. The most common hypothesis explaining these associations between higher animal protein intake and obesity is the effects of amino acids, especially branched-chained amino acids (BCAA) in addition to other amino acids like arginine [8], assumed to stimulate insulin and insulin-like growth factor 1 (IGF-I) secretion as well as affect preadipocyte metabolism leading to overweight in children [9].

Differential DNA methylation might be a mechanism underlying these associations. Associations of early-life dietary intake with DNA methylation have been reported in several studies [10–12]. For example, breastfeeding is associated with early-life growth, with DNA methylation being a potential mediating factor [13–15]. Other studies found associations between the quality and quantity of dietary fat and fatty acids intake and DNA methylation in childhood [16–18].

Quality and quantity of infant protein intake have been previously investigated in association with DNA methylation in animal models [19–21], but never in humans. One study found that maternal protein restriction led to widespread differential methylation and gene expression in new-born rats [22]. Few recent studies found associations between plasma protein metabolites, as an intermediate phenotype of protein intake, and DNA methylation in human adults [23,24].

Better knowledge on possible epigenetic pathways, looking at specific protein sources, might help further understand the underlying mechanisms of early-life dietary programming of later health. Therefore, we examined associations of dietary intake of animal protein in infancy with DNA methylation in early and late childhood. We additionally examined associations of dietary intake of plant protein with DNA methylation at the same time points.

## Materials and methods

### Participants

Data from two studies were used in this meta-analysis; the CHOP trial with participants from five European countries, and the Generation R Study with participants from the Netherlands. The CHOP study is a multi-centre double-blind randomized clinical trial with 1,678 children enrolled in Germany, Belgium, Italy, Poland and Spain. The Generation R Study is a prospective population-based cohort in Rotterdam, the Netherlands. In total, 9,778 mothers were enrolled in the Generation R Study. For CHOP, all children who attended the follow-up visits at ages of 5.5 and 11 years and who agreed on blood withdrawal and DNA methylation measurement were included. In the Generation R Study, a subgroup of children with European ethnic background was selected for DNA methylation measurement at ages of 6 years and 10 years, based on completeness of data. For CHOP, only children who were not breastfed during dietary data collection were included to dietary diary collection. Generation R included only 26 children who were breastfed. In both studies, energy from infant feeding was included in energy intake calculation. Study design and characteristics of both studies have been described previously in detail [25,26]. From both studies, we included participants with information about infant animal protein intake and DNA methylation in early (2–6 years) and/or late childhood (7–12 years). Participants with energy intake from animal and plant protein outside ±5 standard deviations (SDs) from the study mean were excluded from the analysis (*n* = 3 and *n* = 0 in early childhood for CHOP and Generation R, respectively, *n* = 1 and *n* = 0 in late childhood for CHOP and Generation R, respectively). *N* = 183 and *N* = 227 were included in both time points for CHOP and Generation R, respectively. Neither of the studies had siblings in the studied sample. Participants with missing data on any of the covariates were excluded from the analysis (complete case analysis). All children in both studies had European ethnicity. Informed consent was obtained for all participants, and both studies received approval from the local ethics committees.

### Animal and plant protein intake

Animal protein intake was defined as the average daily protein intake from animal food sources, including animal dairy, meat, eggs, and fish combined. Plant protein intake was defined as the average daily protein intake from non-animal sources. Both intakes were measured at approximately 12 and 14 months of age in CHOP and Generation R, respectively. Because we were interested in relative protein intake and to account for confounding by energy intake, we expressed animal protein and plant protein intake as percentage of total energy intake (E%) and additionally included energy intake in our models. In CHOP, dietary intakes were recorded using prospective 3-day dietary diary protocol following standardized operating procedures [27]. Nutrient information was primarily based on the German national food composition database (BLS 3.01). Food items and recipes not identified in the database were added by CHOP dietitians at each study centre according to information from the manufacturers, other databases or ingredients. Food records with energy or macronutrient intakes > 3 SDs of the mean by month and country and those noted by the data entering dietitian to be incomplete or inaccurate or with reported concurrent illness were excluded [28]. In the Generation R Study, dietary intake was evaluated using a 211-item semi-quantitative Food Frequency Questionnaire (FFQ) covering the previous month. The Dutch Food Composition Table 2006 and standard Dutch portion sizes were used to convert food frequencies into energy and macronutrient intakes [29].

### DNA methylation data acquisition, quality control, and normalization

DNA was isolated from peripheral whole blood samples in both studies, using the salting out method. For early childhood, the average age at blood draw was 5.5 and 6 years for CHOP and Generation R, respectively, while it was 11 and 10 years for CHOP and Generation R, respectively, for late childhood. For CHOP and Generation R, 800 and 500 ng of DNA, respectively, per sample underwent bisulphite conversion. DNA methylation in early and late childhood was measured using the Illumina Infinium® HumanMethylation450 BeadChip assay. In both studies, DNA methylation data normalization and quality control were done following the quantile normalization method (CPACOR) by Lehne et. al [30]. DNA methylation was analysed as untransformed DNA methylation beta-values ranging from 0 (completely unmethylated) to 1 (completely methylated) expressing the proportion of cells in which the DNA was methylated at a specific cytosine-phosphate-guanine (CpG) site. Non-autosomal probes were excluded. Control probes were removed as part of study-level QC performed on DNA methylation data. The top and bottom 0.5% methylation beta-values were winsorized.

### Covariates

Models were adjusted for total energy intake (from FFQ or dietary protocol), age at blood collection, sex, self-reported highest completed maternal educational level (lower/higher), gestational age at birth, self-reported maternal pre-pregnancy BMI, self-reported sustained maternal smoking during pregnancy into at least the second trimester (yes/no), blood cell proportions (B-cells, CD8+ T-cells, CD4+ T-cells, granulocytes, NK-cells, and monocytes) estimated using the reference-based Houseman method [31] in the minfi package in R [32,33], with the Reinius reference [34] in both studies. To control for technical batch effects, both studies adjusted for sample plate number as a technical covariate. For CHOP, the country of study centre was also included in all models.

### Statistical analysis

Robust linear regression models were run in both studies to analyse the associations of both infant animal and plant protein intake and DNA methylation at both time points following a pre-specified analysis plan and R code. For animal protein intake, each study ran eight robust linear regression models (four models at each of the two time points) using the rlm() function of the ‘MASS’ package in R [33,35], with model 4 (fully adjusted model) being the main model. For covariance matrix estimation, ‘Heteroscedasticity-Consistent Covariance Matrix Estimation’ method was used. For plant protein intake, each study ran the fully adjusted model at each time point. To account for confounding by energy intake, the multivariate nutrient density model was used, expressing animal protein in E% and additionally including energy intake as covariate:

1. DNAm ~ Infant animal/plant protein intake + age at blood collection + sex + technical covariates
2. Model 1 plus adjustment for total energy intake
3. Model 2 plus adjustment for maternal educational level + gestational age at birth + pre-pregnancy BMI + maternal smoking during pregnancy
4. Model 3 plus adjustment for cell counts

To identify sex-specific DNA methylation differences, we also performed sex-stratified meta-analyses for both animal and plant protein intake at both time points using the fully adjusted model 4. Before meta-analysis, quality control was done on the results of both studies using the QCEWAS R package [36]. Results from both studies were centrally meta-analysed using METAL [37] by MeS, using fixed effects inverse variance-weighted meta-analysis in METAL. The meta-analysis was then independently repeated by JFF, to exclude the possibility of human error. Removing cross-reactive probes [38,39] (*N* = 43,635 in early childhood and *N* = 43,702 in late childhood) and probes available only in one study (*N* = 13,428 in early childhood and *N* = 14,799 in late childhood) resulted in 415,274 and 414,307 probes included in the meta-analyses for early and late childhood, respectively. We flagged CpGs potentially influenced by a single nucleotide polymorphism (polymorphic sites) [38,39] and CpGs listed as methylation quantitative trait loci (mQTLs) [40]. Hartigans’ dip test for unimodality was used to check if methylation level at significant CpGs was influenced by a nearby single nucleotide polymorphism. We also flagged CpGs with high between-study heterogeneity, defined as an I^2^ >50%. We decided *a priori* on a false discovery rate (FDR)-adjusted p-value significance level of < 0.05 [41]. All statistical analyses were performed using R statistical analysis software [33], except for the meta-analysis in METAL.

### Additional analyses

Following the meta-analyses, multiple analyses were performed on the top CpGs (FDR *P* < 0.05) from the fully adjusted non sex-stratified meta-analyses at both time points, to examine potential functional consequences. First, the EWAS catalog [42] was used to identify previously published associations with other traits for the top CpGs. Second, we checked if the top CpGs were previously reported to be associated with gene expression in childhood blood, measured as expression quantitative trait methylation (eQTM). For this, we used the catalogue of 13.6 million blood autosomal cis-eQTMs in children published by the Human Early Life Exposome (HELIX) project, after cell type adjustment [43]. Third, we ran functional enrichment analyses using Gene Ontology (GO) and Kyoto Encyclopedia of Genes and Genomes (KEGG) in the MissMethyl R package [44] on all CpGs with *P* < 1×10¯^4^ in the fully adjusted models at both time points for both animal and plant protein intakes.

## Results

### Participants

In total, 579 and 604 children were included in the animal protein meta-analyses and 577 and 604 in the plant protein meta-analysis for early and late childhood, respectively. Study-specific descriptives are presented in Tables 1 and 2. Infant animal and plant protein intakes were normally distributed in both studies. Animal protein had means (SD) of 10.6% (3.2) and 10.7% (3.5) in CHOP for the analyses in early and late childhood DNA methylation, respectively, and 8.1% (2.4) and 8.1% (2.4) in Generation R for analyses in early and late childhood DNA methylation, respectively. Plant protein intake had means (SD) of 4.04 (1.03) and 3.93 (1.06) in CHOP for the analyses in early and late childhood DNA methylation, respectively, and 4.67 (1.31) and 4.76 (1.26) in Generation R for analyses in early and late childhood DNA methylation, respectively (Supplementary Figures S1A-1D and 2A-2D).

**Table 1.** Study-specific descriptives for animal protein analysis.

	Early childhood DNAm measurement		Late childhood DNAm measurement	
	CHOP N = 255	Generation R N = 324	CHOP N = 300	Generation R N = 304
**E% from animal protein** (%)	10.6 (3.2)	8.1 (2.4)	10.7 (3.5)	8.1 (2.4)
**Total energy intake** (kcal)	894 (169)	1309 (331)	887 (167)	1322 (334)
**Age at blood collection** (years)	5.6 (0.1)	6.0 (0.2)	11.3 (0.13)	9.78 (0.24)
**Age at dietary data collection** (months)	11.9 (0.2)	13.8 (1.7)	11.9 (0.2)	14.0 (1.8)
**Sex**				
Boy	120 (47.1%)	154 (47.5%)	147 (49.0%)	157 (51.6%)
Girl	135 (52.9%)	170 (52.5%)	153 (51.0%)	147 (48.4%)
**Maternal smoking during pregnancy**				
No smoking/smoking stopped before 2nd trimester	217 (85.1%)	290 (89.5%)	252 (84.0%)	270 (88.8%)
Sustained smoking into 2nd trimester	38 (14.9%)	34 (10.5%)	48 (16.0%)	34 (11.2%)
Maternal educational level*				
Low	45 (17.6%)	82 (25.3%)	51 (17.0%)	88 (28.9%)
High	210 (82.4%)	242 (74.7%)	249 (83.0%)	216 (71.1%)
**Maternal BMI** (kg/m^2^)	23.9 (4.1)	23.1 (3.6)	23.6 (3.8)	23.2 (3.6)
**Gestational age at birth** (weeks)	39.7 (1.2)	40.3 (1.4)	39.8 (1.2)	40.2 (1.4)
**CHOP Study centre**				
Belgium	43 (16.9%)	−	36 (12.0%)	−
Spain	89 (34.9%)	−	101 (33.7%)	−
Germany	18 (7.1%)	−	42 (14.0%)	−
Italy	105 (41.2%)	−	91 (30.3%)	−
Poland	−	−	30 (10.0%)	−

DNAm= DNA methylation.

Results presented as mean ± SD or N (%).

*In both cohorts, maternal educational level was defined as ‘Low’ if no, primary or secondary school education obtained, and as ‘High’ if college or university degree obtained.

**Table 2.** Study-specific descriptives for plant protein analysis.

	Early childhood DNAm measurement		Late childhood DNAm measurement	
	CHOP N = 255	Generation R N = 322	CHOP N = 300	Generation R N = 302
**E% from plant protein** (%)	4.04 (1.03)	4.67 (1.31)	3.93 (1.06)	4.76 (1.26)
**Total energy intake** (kcal)	894 (169)	1311 (331)	887 (167)	1323 (333)
**Age at blood collection** (years)	5.6 (0.1)	6.0 (0.2)	11.3 (0.13)	9.77 (0.24)
**Age at dietary data collection** (months)	11.9 (0.2)	13.8 (1.7)	11.9 (0.2)	14.0 (1.8)
**Sex**				
Boy	120 (47.1%)	154 (47.8%)	147 (49.0%)	157 (52.0%)
Girl	135 (52.9%)	168 (52.2%)	153 (51.0%)	145 (48.0%)
**Maternal smoking during pregnancy**				
No smoking/smoking stopped before 2nd trimester	217 (85.1%)	288 (89.4%)	252 (84.0%)	268 (88.7%)
Sustained smoking into 2nd trimester	38 (14.9%)	34 (10.6%)	48 (16.0%)	34 (11.3%)
Maternal educational level*				
Low	45 (17.6%)	82 (25.5%)	51 (17.0%)	88 (29.1%)
High	210 (82.4%)	240 (74.6%)	249 (83.0%)	214 (70.9%)
**Maternal BMI** (kg/m^2^)	23.9 (4.1)	23.1 (3.6)	23.6 (3.8)	23.2 (3.6)
**Gestational age at birth** (weeks)	39.7 (1.2)	40.3 (1.4)	39.8 (1.2)	40.2 (1.4)
**CHOP Study centre**				
Belgium	43 (16.9%)	−	36 (12.0%)	−
Spain	89 (34.9%)	−	101 (33.7%)	−
Germany	18 (7.1%)	−	42 (14.0%)	−
Italy	105 (41.2%)	−	91 (30.3%)	−
Poland	−	−	30 (10.0%)	−

DNAm= DNA methylation.

Results presented as mean ± SD or N (%).

*In both cohorts, maternal educational level was defined as ‘Low’ if no, primary or secondary school education obtained, and as ‘High’ if college or university degree obtained.

### Meta-analysis

**Figure 1.**
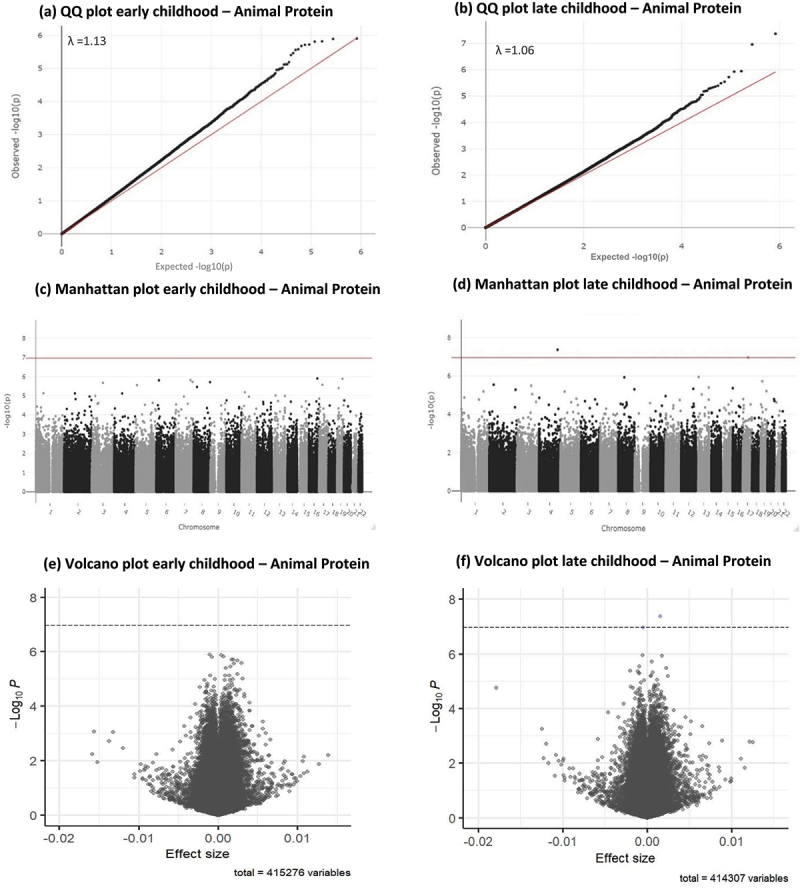
Q-Q, Manhattan and volcano plots for associations between infant animal protein intake (E%) and early and late childhood DNA methylation. Significance line in Manhattan and volcano plots is set to represent the Bonferroni-corrected p-value threshold of *P* < 1.1 × 10¯^7^.

**Figure 2.**
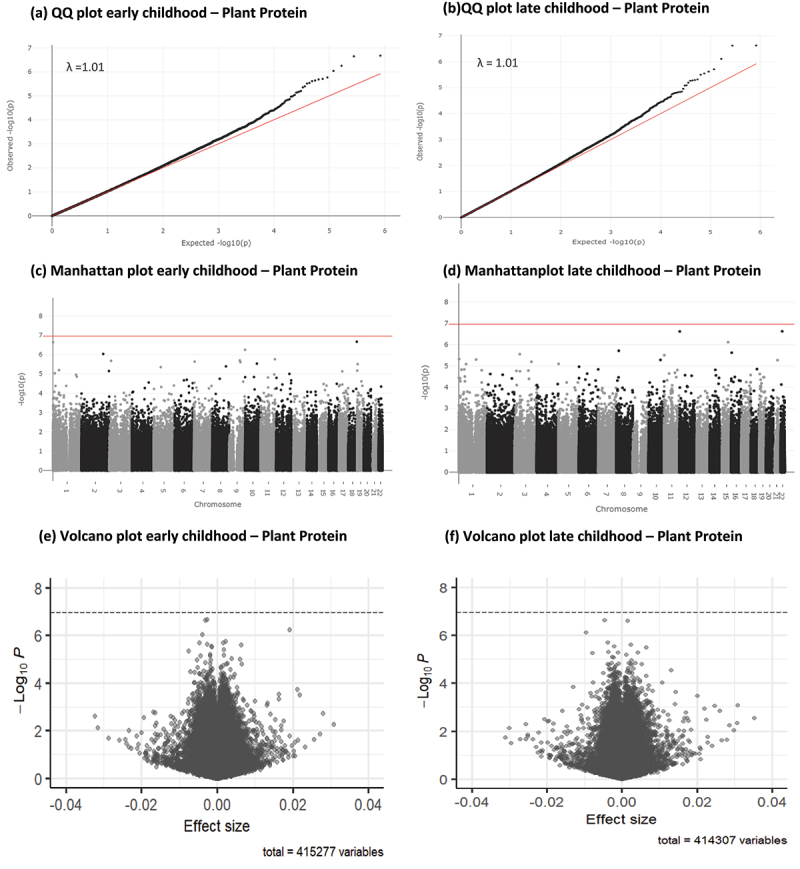
Q-Q, Manhattan and volcano plots for associations between infant plant protein intake (E%) and early and late childhood DNA methylation. Significance line in Manhattan and volcano plots is set to represent the Bonferroni-corrected p-value threshold of *P* < 1.1 × 10¯^7^.

Results of the epigenome-wide association study (EWAS) meta-analysis for the fully adjusted models (model 4) at both time points, for CpGs with *P* < 1×10¯^5^ are presented in Tables 3 and 4 for animal protein and in Tables 5 and 6 for plant protein. Sex-stratified EWAS results are presented in Supplementary tables S1-S8. Full EWAS meta-analysis results are available via: https://doi.org/10.5281/zenodo.8375454. Q-Q, Manhattan and Volcano plots for the meta-analysis results of the fully adjusted model at both time points are presented in Figure 1a–f for animal protein and Figure 2a–f for plant protein. Cohort-specific Q-Q plots and lambdas are shown in Supplementary Figures S3A-3D for animal protein and S4A-4D for plant protein. Q-Q plots for sex-stratified meta-analysis results are shown in Supplementary figures S5A-5D for animal protein and Supplementary figures S6A-6D for plant protein. Infant animal protein intake was not associated with DNA methylation at any CpG site in early childhood, after FDR correction.

**Table 3.** Meta-analysis results for associations of infant animal protein intake (E%) and early childhood whole blood DNA methylation levels at *P* < 1 × 10¯^5.^.

CpG	Effect^a^	SE^a^	P value	FDR	Direction^b^	I^2^	Polymorphic	mQTL^c^	Chr	Position	Relation to Island	Nearest Gene	Gene Region
cg02751838	−0.10	0.02	1.25E–06	0.12	- -	48.7	Yes	Yes	16	75148330	N_Shore	*LDHD*	Body
cg19657814	0.04	0.01	1.29E–06	0.12	+ +	13.4	No	Yes	19	47137444	N_Shore	*GNG8*	TSS1500
cg14183329	0.06	0.01	1.53E–06	0.12	+ +	53.9	Yes	Yes	7	131242962	Island	*PODXL*	TSS1500
cg11780382	−0.08	0.02	1.55E–06	0.12	- -	54.8	No	Yes	6	24719812	N_Shore	*C6orf62*	-
cg18177414	0.19	0.04	1.90E–06	0.12	+ +	73	No	Yes	7	149389929	Island	*KRBA1*	-
cg24044478	0.17	0.04	1.94E–06	0.12	+ +	0	No	Yes	8	145035191	OpenSea	*PLEC*	Body
cg04730047	0.24	0.05	2.09E–06	0.12	+ +	0	No	Yes	3	99979355	N_Shore	*TBC1D23*	Body
cg20469139	0.30	0.06	2.64E–06	0.12	+ +	0	No	Yes	17	29297458	N_Shore	*RNF135*	TSS1500
cg27639457	0.20	0.04	2.76E–06	0.12	+ +	0	No	Yes	5	11384753	Island	*CTNND2*	Body
cg05226685	0.15	0.03	3.46E–06	0.14	+ +	0	Yes	No	8	30010237	N_Shelf	*MIR548O2*	-
cg10667167	0.10	0.02	3.97E–06	0.15	+ +	38.3	No	No	19	12662910	S_Shore	*ZNF564*	TSS1500
cg08785524	0.24	0.05	6.49E–06	0.21	+ +	0	No	No	11	29340853	OpenSea	*KCNA4*	-
cg04723723	0.09	0.02	7.50E–06	0.21	+ +	0	No	No	1	67966270	OpenSea	*SERBP1*	-
cg08468732	0.14	0.03	7.54E–06	0.21	+ +	0	No	Yes	2	95722029	OpenSea	*MAL*	-
cg23991274	−0.10	0.02	7.61E–06	0.21	- -	68.3	No	No	4	71860600	S_Shore	*DCK*	Body
cg08756033	0.31	0.07	9.65E–06	0.22	+ +	85.5	Yes	Yes	13	31480128	N_Shore	*TEX26-AS1*	TSS200

^a^Effect sizes and SE are presented as a percent change in DNA methylation per 1 E% increase in energy from animal protein intake.

^b^Studies are arranged from left to right as follows: CHOP then Generation R.

^c^methylation quantitative trait loci (mQTL) utilized from GoDMC database.

EWAS model was adjusted for total energy intake, age at blood collection, sex, maternal educational level, gestational age, pre-pregnancy BMI, maternal smoking during pregnancy, technical covariates, cell counts.

**Table 4.** Meta-analysis results for associations of infant animal protein intake (E%) and late childhood whole blood DNA methylation levels at *P* < 1 × 10¯^5.^.

CpG	Effect^a^	SE^a^	P value	FDR	Direction^b^	I^2^	Polymorphic	mQTL^c^	Chr	Position	Relation to Island	Nearest Gene	Gene Region
cg21300373	0.16	0.03	4.27E–08	0.018	+ +	0	No	Yes	4	165304540	Island	*MARCHF1*	TSS200
cg10633363	−0.05	0.01	1.09E–07	0.022	- -	50.6	No	Yes	17	46703854	Island	*HOXB9*	TSS200
cg10167561	−0.06	0.01	1.13E–06	0.122	- -	0	Yes	No	13	22244956	Island	*FGF9*	TSS1500
cg15680470	0.17	0.04	1.18E–06	0.122	+ +	49.5	Yes	No	8	54938360	S_Shelf	*TCEA1*	-
cg09463900	0.05	0.01	1.90E–06	0.157	+ +	0	No	No	19	12939587	Island	*RTBDN*	Body
cg27026673	−0.10	0.02	2.85E–06	0.180	- -	29.9	No	Yes	2	39664343	Island	*MAP4K3*	TSS200
cg16516295	0.23	0.05	3.25E–06	0.180	+ +	0	No	Yes	3	129147846	S_Shore	*EFCAB12*	TSS1500
cg26730050	−0.20	0.04	4.04E–06	0.180	- -	27.2	No	Yes	13	112655007	OpenSea	*SOX1*	-
cg06993367	−0.11	0.02	4.36E–06	0.180	- -	0	No	Yes	16	982031	S_Shore	*LMF1*	Body
cg06516150	0.06	0.01	4.68E–06	0.180	+ +	0	No	Yes	12	109592528	Island	*ACACB*	Body
cg04621866	−0.10	0.02	4.97E–06	0.180	- -	0	No	No	8	144567481	N_Shore	*ZC3H3*	Body
cg13982505	−0.10	0.02	5.20E–06	0.180	- -	40.5	No	Yes	2	233642094	OpenSea	*GIGYF2*	TSS1500, Body
cg02288564	0.05	0.01	6.22E–06	0.182	+ +	0	No	No	19	49934404	Island	*SLC17A7*	Body
cg12140668	0.04	0.01	6.46E–06	0.182	+ -	84.5	No	No	1	197744252	Island	*DENND1B*	Body
cg15355952	0.24	0.05	6.60E–06	0.182	+ +	0	No	Yes	5	36662829	OpenSea	*SLC1A3*	Body
cg05965863	0.12	0.03	8.90E–06	0.222	+ +	0	No	Yes	3	137480182	Island	*SOX14*	-
cg21074631	−0.04	0.01	9.12E–06	0.222	- -	0	No	No	13	50422213	Island	*CTAGE10P*	-

^a^Effect sizes and SE are presented as a percent change in DNA methylation per 1 E% increase in energy from animal protein intake.

^b^Studies are arranged from left to right as follows: CHOP then Generation R.

^c^methylation quantitative trait loci (mQTL) utilized from GoDMC database.

EWAS model was adjusted for total energy intake, age at blood collection, sex, maternal educational level, gestational age, pre-pregnancy BMI, maternal smoking during pregnancy, technical covariates, cell counts.

**Table 5.** Meta-analysis results for associations of infant plant protein intake (E%) and early childhood whole blood DNA methylation levels at *P* < 1 × 10¯^5.^.

CpG	Effect^a^	SE^a^	P value	FDR	Direction^b^	I^2^	Polymorphic	mQTL^c^	Chr	Position	Relation to Island	Nearest Gene	Gene Region
cg15407373	−0.003	0.0005	2.13E–07	0.047	- -	95.6	No	Yes	18	74800029	Island	*MBP*	Body
cg25973293	−0.003	0.0006	2.26E–07	0.047	- -	5.2	No	Yes	1	1061647	OpenSea	*C1orf159*	-
cg15473904	0.019	0.0038	5.62E–07	0.078	+ +	73.3	No	Yes	9	140446993	S_Shore	*MRPL41*	3’UTR
cg23235135	−0.004	0.0008	9.22E–07	0.096	- -	39.0	No	Yes	2	189850571	OpenSea	*COL3A1*	Body
cg03460239	0.002	0.0005	1.73E–06	0.113	+ +	71.8	No	No	11	124311153	OpenSea	*OR8B8*	TSS200
cg13920278	−0005	0.0010	1.98E–06	0.113	- -	0	No	Yes	9	93852285	OpenSea	*LOC100129316*	-
cg03870270	0.002	0.0003	2.08E–06	0.113	+ +	0	No	No	3	15643045	Island	*HACL1*	1stExon; TSS1500
cg14784944	−0003	0.0006	2.30E–06	0.113	- -	0	No	Yes	7	4855785	N_Shore	*RADIL*	Body
cg13558954	0.006	0.0013	2.45E–06	0.113	+ -	75.4	Yes	No	9	99521386	OpenSea	*ZNF510*	Body
cg18861311	−0.002	0.0003	2.92E–06	0.117	- -	54.4	No	Yes	10	102747418	Island	*TWNK*	1stExon; TSS200
cg23290217	−0.001	0.0003	3.09E–06	0.117	- -	0	No	Yes	19	4909290	Island	*UHRF1*	TSS1500
cg05040429	0.002	0.0004	4.05E–06	0.140	+ +	78.7	No	No	8	119633844	N_Shore	*SAMD12*	Body
cg01233720	−0.008	0.0016	4.45E–06	0.142	- -	0	Yes	Yes	5	60456037	N_Shore	*SMIM15*	5’UTR
cg23272399	−0.002	0.0004	6.32E–06	0.180	- -	81.1	No	Yes	1	53068579	Island	*GPX7*	Body
cg26753088	0.002	0.0004	6.74E–06	0.180	+ +	75.0	No	Yes	19	1293582	N_Shore	*EFNA2*	Body
cg02006854	−0.002	0.0004	7.09E–06	0.180	- -	0	Yes	No	2	239542460	Island	*LINC01107*	-
cg18459618	−0.004	0.0010	7.38E–06	0.180	- -	78.6	No	Yes	17	29297478	N_Shore	*RNF135*	TSS1500
cg06747907	0.004	0.0009	9.22E–06	0.213	+ +	0	No	Yes	1	16091100	OpenSea	*FBLIM1*	TSS1500; 1stExon
cg19685229	0.006	0.0014	9.94E–06	0.216	+ +	0	No	No	12	116587387	OpenSea	*MED13L*	TSS1500; Body

^a^Effect sizes and SE are presented as a percent change in DNA methylation per 1 E% increase in energy from animal protein intake.

^b^Studies are arranged from left to right as follows: CHOP then Generation R.

^c^methylation quantitative trait loci (mQTL) utilized from GoDMC database.

EWAS model was adjusted for total energy intake, age at blood collection, sex, maternal educational level, gestational age, pre-pregnancy BMI, maternal smoking during pregnancy, technical covariates, cell counts.

**Table 6.** Meta-analysis results for associations of infant plant protein intake (E%) and late childhood whole blood DNA methylation levels at *P* < 1 × 10¯^5.^.

CpG	Effect^a^	SE^a^	P value	FDR	Direction^b^	I^2^	Polymorphic	mQTL^c^	Chr	Position	Relation to Island	Nearest Gene	Gene Region
cg06623197	−0.005	0.001	2,39E–07	0.050	- -	85.1	No	Yes	22	30400763	OpenSea	*MTMR3*	Body
cg16739865	0.001	0.000	2,43E–07	0.050	+ +	70.6	No	No	12	3186281	Island	*TSPAN9*	TSS1500
cg18106898	−0.010	0.002	7,79E–07	0.108	- -	0	No	Yes	15	74229671	OpenSea	*LOXL1*	Body
cg26954671	−0.004	0.001	1,97E–06	0.188	- -	0	Yes	No	8	22223465	N_Shore	*SLC39A14*	TSS1500
cg03368634	0.002	0.000	2,42E–06	0.188	+ +	0	No	No	16	3824553	OpenSea	*CREBBP*	Body
cg06333135	−0.002	0.000	2,84E–06	0.188	- -	24.2	No	No	3	47460006	Island	*SCAP*	Body
cg20272155	0.006	0.001	3,17E–06	0.188	+ +	38	No	Yes	11	1769462	Island	*MOB2*	Body; TSS200
cg01104489	−0.004	0.001	4,84E–06	0.204	- -	27.2	No	Yes	1	3072235	Island	*PRDM16*	Body
cg04450606	0.004	0.001	5,08E–06	0.204	+ +	54.4	Yes	No	1	1,53E + 08	OpenSea	*PGLYRP3*	TSS200
cg12198729	−0.001	0.000	5,28E–06	0.204	- -	15.6	No	No	10	1,03E + 08	Island	*BTRC*	TSS200; TSS200
cg01668281	−0.008	0.002	5,41E–06	0.204	- -	0	No	Yes	21	37915281	OpenSea	*CLDN14*	5’UTR; TSS1500
cg24353466	0.001	0.000	6,42E–06	0.222	+ +	0	No	No	3	1,5E + 08	Island	*RNF13*	1stExon; 5’UTR
cg21953251	−0.003	0.001	8,13E–06	0.245	- -	51.5	Yes	No	5	766985	OpenSea	*ZDHHC11*	-
cg15209277	−0.002	0.000	8,29E–06	0.245	- -	0	No	No	1	64472035	OpenSea	*ROR1*	Body

^a^Effect sizes and SE are presented as a percent change in DNA methylation per 1 E% increase in energy from animal protein intake.

^b^Studies are arranged from left to right as follows: CHOP then Generation R.

^c^methylation quantitative trait loci (mQTL) utilized from GoDMC database.

EWAS model was adjusted for total energy intake, age at blood collection, sex, maternal educational level, gestational age, pre-pregnancy BMI, maternal smoking during pregnancy, technical covariates, cell counts.

Infant animal protein intake was statistically significantly associated with DNA methylation in late childhood at two CpG sites: cg21300373 (0.16% increase in DNA methylation per each 1 E% increase in infant animal protein intake, SE = 0.0003, *P* = 4.27×10^−8^, *P*_*FDR*_ = 0.018, I^2^ = 0) and cg10633363 (0.05% decrease in DNA methylation per each 1 E% increase in infant animal protein intake, SE = 0.0001, *P* = 1.09×10^−7^, *P*_*FDR*_ = 0.022, I^2^ = 51) after FDR correction. Forest plots for both CpGs associated with animal protein intake are presented in Supplementary figures S7 and 8 and Supplementary figures S9 and 10 for plant protein. Even though cg10633363 had an I^2^ of 50.6, the forest plots did not show clear heterogeneity between studies. Both CpGs were flagged as potentially associated with a SNP (mQTLs). Although the density plots showed a slight indication of multimodality (Supplementary figures S11 and 12), Hartigans’ dip test had a *P* > 0.05 for both CpGs in both studies, indicating no significant deviation from unimodality.

To check for consistency of results across both time points, we conducted a look-up in the early childhood results of the two CpGs associated with animal protein in late childhood. Neither of the CpGs, cg21300373 (Effect = 0.0006, SE = 0.0004, *P* = 0.13, *P*_*FDR*_ = 0.84, I^2^ = 0) and cg10633363 (Effect = 0.0001, SE = 0.0002, *P* = 0.70, *P*_*FDR*_ = 0.97, I^2^ = 21), was significantly associated with animal protein intake in early childhood. Also, for CpGs associated with plant protein in early childhood, we conducted a look-up in the late childhood EWAS results. Neither of the CpGs, cg15407373 (Effect= −0.0001, SE = 0.0006, *P* = 0.80, *P*_*FDR*_ = 1.00, I^2^ = 0) and cg25973293 (Effect= −0.0009, SE = 0.0005, *P* = 0.09, *P*_*FDR*_ = 0.96, I^2^ = 0), were significantly associated with plant protein intake in late childhood (Supplementary table S9).

Infant plant protein intake was associated with DNA methylation after FDR correction at 2 CpG sites: cg25973293 (0.003% decrease in DNA methylation per each 1 E% increase in infant plant protein intake, SE = 0.0006, *P* = 2.26×10^−7^, *P*_*FDR*_ = 0.047, I^2^ = 5.2) and cg15407373 (0.003% decrease in DNA methylation per each 1 E% increase in infant plant protein intake, SE = 0.0005, *P* = 2.13×10^−7^, *P*_*FDR*_ = 0.047, I^2^ = 95.6) in early childhood. Cg25973293 is flagged as potentially associated with an mQTL.

Plant protein intake was not associated with DNA methylation at any CpG site in late childhood, after FDR correction. Neither of the two CpGs associated with animal protein intake were associated with plant protein intake at *P* < 1×10¯^5^, or vice versa.

Sex-stratified meta-analyses showed sex-specific DNA methylation differences in both early and late childhood with infant animal protein intake. Infant animal protein intake was associated with early childhood DNA methylation at 16 CpG sites in boys and at 9 CpG sites in girls, after FDR correction. Infant animal protein intake was associated with late childhood DNA methylation at 11 CpG sites in boys and at 1 CpG site in girls, after FDR correction. Cg21300373, one of the CpGs significantly associated with animal protein in late childhood in both sexes combined, was only significant in boys (Effect = 0.002, SE = 0.0004, *P* = 1.24×10^−6^, *P*_*FDR*_ = 0.046, I^2^ = 3.9) and not in girls (Effect = 0.001, SE = 0.0004, *P* = 7.6×10^−4^, *P*_*FDR*_ = 0.34, I^2^ = 0), after FDR correction. Cg10633363, the other CpG significantly associated with animal protein in late childhood in both sexes combined, was not significant neither in boys (Effect = 0.001, SE = 0.0003, *P* = 2.41×10^−4^, *P*_*FDR*_ = 0.34, I^2^ = 0) nor in girls (Effect = 0.001, SE = 0.0003, *P* = 1.17×10^−5^, *P*_*FDR*_ = 0.14, I^2^ = 57) (Supplementary tables S1-S4).

For infant plant protein intake, sex-stratified meta-analysis showed associations with DNA methylation in early childhood at 1 CpG site for boys and at 3 CpG sites for girls, respectively. Associations of infant plant protein intake with DNA methylation at late childhood were found at 9 CpG sites for boys and at 1 CpG site for girls, respectively. None of the findings in the sex-stratified plant protein meta-analyses was found in the other sex or in any of the other sex-stratified models (Supplementary tables S5-S8).

### Functional analysis

Cg21300373 is mapped to *MARCHF1* and cg10633363 is mapped to *HOXB9*, both CpGs are located in the transcription start site. We used the CpGs associated with animal protein for functional analyses. First, a look-up in the EWAS catalog showed that DNA methylation at cg21300373 was previously associated with pancreatic ductal adenocarcinoma and clear cell renal carcinoma [45,46]. Cg10633363 was not reported in the EWAS catalog. Second, cg21300373 and cg10633363 were not associated with gene expression in the catalogue of blood autosomal cis-eQTMs in children [47]. Third, functional enrichment analysis was done using the CpGs with p-values <1×10¯^4^. For animal protein models, 120 CpGs for early childhood and 98 CpGs for late childhood models were used as input. For plant protein, 88 CpGs for early childhood and 89 CpGs for late childhood were used as input. There were no enriched GO or KEGG pathways (FDR <0.05) at either animal or plant protein intake at either time point. Cg25973293 associated with infant plant protein intake at early childhood is mapped to *C1orf159*. Cg25973293 was not reported to be associated with other phenotypes in the EWAS catalog.

## Discussion

In this meta-analysis, infant animal protein intake was associated with DNA methylation in late childhood at the age of 7–12 years at two CpG sites; cg21300373 and cg10633363, but not with DNA methylation in early childhood. No associations with specific functional pathways or gene expression were identified. Infant plant protein intake was associated with DNA methylation in early childhood at the age of 2–6 years at two CpG sites; cg25973293 and cg15407373.

Cg21300373 was mapped to the transcription start site of *MARCHF1* which is a member of the *MARCH* family of membrane-bound E3 ubiquitin ligases and was linked to glucose-tolerance and lipid storage [48]. E3 ubiquitin ligases play a pivotal role in obesity-induced insulin resistance in humans [49]. DNA methylation of *MARCHF1* gene was found to be associated with adiposity in a previous EWAS in multi-ethnic Asian adults [50]. *MARCHF1* genetic variants have also been associated with adiposity in adults [51,52]. Therefore, this may be a first link between protein intake-related DNA methylation and obesity in children.

Cg10633363 was mapped to the transcription start site of *HOXB9*. *HOXB9* is one of the *HOX* genes, a group of related genes responsible for mapping body organs along the head-tail axis during embryonic development. *HOXB9* has not been linked to body composition. DNA methylation levels at CpG sites in or close to *HOXB9* have been found to be associated with epigenetic ageing of liver tissue [53], a process that was previously found to be accelerated by obesity [54].

Cg25973293, associated with plant protein, is mapped to *C1orf159* gene in an open sea. *C1orf159* is a protein coding gene whose increased expression was found to be an unfavourable prognostic marker in renal [55] and liver [56] cancer. Despite being significant after FDR correction, cg15407373 had heterogeneity I^2^ = 95.6 denoting high heterogeneity between results from both analysed studies results. Cg15407373, associated with plant protein, is mapped to *MBP* gene in an island. *MBP* gene is a protein coding gene responsible for encoding Myelin Basic Protein, a protein playing a pivotal role in nervous system development [57].

No associations of genetic variants of *HOXB9, C1orf159* and *MBP* genes with metabolic phenotypes have been reported in the GWAS catalog [58]. Although all CpGs discovered in animal protein and plant protein EWAS are listed to be associated with a genetic variant, the level of methylation at these sites did not seem to be strongly influenced by a nearby polymorphic site in our populations, as the distribution of the methylation values for both sites in both studies did not differ significantly from unimodality. Using CpGs associated with infant animal and plant protein intakes with *P* < 1×10¯^4^ for early and late childhood models, no enriched functional pathways or biological processes were found. Further research is needed to confirm any mediating role for DNA methylation in the associations of animal protein intake and health in later life. The infant plant protein intake EWAS showed no association for the two CpGs discovered in infant animal protein EWAS with DNA methylation at either of the two time points investigated. Sex-specific DNA methylation differences were identified at both time points for both infant animal and plant protein intakes.

Previous studies showed associations between early life protein intake, especially animal protein, and body composition, mainly higher fat mass [59]. Although studies in animal models have reported that quantity and quality of protein intake in pups are associated with DNA methylation [21,60], whether this may be a potential underlying mechanism in the association of protein with adiposity has not been investigated in humans [4,5,61].

Perinatal exposures, such as early nutrition, are important for early health programming. It is also becoming clear that nutritional associations with DNA methylation are not limited to perinatal period, but further extend to postnatal life [62]. Infant breastfeeding was found to be associated with child’s growth and development, with DNA methylation being a potential mediating factor [14]. Our study contributes to this evidence, linking infant animal protein intake with DNA methylation. These associations appeared only later in childhood, suggesting an association with extended exposure. Furthermore, different associations were found for infant animal and plant protein intakes, suggesting a role for the difference in metabolism of animal and plant protein. The quality of protein and availability of methyl group donors might play a role in differential methylation of some CpG sites. Animal protein as a nutritional source is rich in methionine [63], a precursor to the universal methyl-donor S-adenosylmethionine, which is assumed to change the DNA methylation pattern, especially at specific loci rather than genome-wide [64].

Sex-specific differences were observed for infant animal and plant protein intakes which might be attributed to disparities in protein metabolism between the sexes [59,65]. Further research including more studies at multiple time points might better reveal the association of animal protein intake and DNA methylation throughout the life course and whether associations of animal protein with DNA methylation indeed mediate associations with body composition.

This study has a number of strengths. We defined a strict age window for infant animal and plant protein intakes, because dietary composition in early life can change drastically over time [66]. Both cohorts included in this meta-analysis have detailed dietary data, as well as information on relevant confounders. The analyses were run using a pre-specified analysis plan. We included mother-offspring data from multiple European countries. The multivariate nutrient density model was used to account for total energy intake [67]. However, this study has a limited sample size, which may have limited our ability to find smaller effect sizes. As macronutrient intake is known to be correlated with total energy intake, analyses may be confounded by total energy intake [67], to overcome this, we adjusted for total energy intake in the main models while using E% from animal protein sources as the exposure. With this adjustment we can interpret the results as the effect of isocaloric replacement of animal protein with any other macronutrient. Children from both cohorts were of European ethnicity which might limit generalizability of the findings to other ethnic groups, however it reduces the heterogeneity between the two studies included. Both cohorts relied on self-reported dietary assessment questionnaires, which might have introduced measurement error. Despite both cohorts using different dietary assessment methods, forest plots for significant CpGs showed consistent results from both cohorts with slightly different effect sizes, which might be a reason for the marginally high I^2^ (50.6%) for cg10633363 [68].

In conclusion, this meta-analysis showed associations of infant animal protein intake with DNA methylation at cg21300373 and cg10633363 in late childhood. No associations were found between infant animal protein intake and DNA methylation in early childhood. Association of infant plant protein intake with DNA methylation was found at cg25973293 and cg15407373 in early childhood. No associations were found between plant protein and DNA methylation in late childhood. Potential mediating epigenetic pathways between infant protein intake and health outcomes require further investigation using larger sample sizes.

## Supplementary Material

Supplementary.docx

## Data Availability

The databases used here in this study were exclusively available for the authors to access and use after an administrative permission. All relevant data and analysis code used in supporting the findings in this research article can be made available upon a reasonable request. Full EWAS results for all models can be found at: https://doi.org/10.5281/zenodo.8375454.

## References

[1] Michaelsen KF, Greer FR. Protein needs early in life and long-term health. Am J Clin Nutr. 2014;99(3):718s–15. doi: 10.3945/ajcn.113.072603 24452233

[2] Lind MV, Larnkjær A, Mølgaard C, et al. Dietary protein intake and quality in early life: impact on growth and obesity. Curr Opin Clin Nutr Metab Care. 2017;20(1):71–6. doi: 10.1097/MCO.0000000000000338 27749711

[3] Jen V, Braun KVE, Karagounis LG, et al. Longitudinal association of dietary protein intake in infancy and adiposity throughout childhood. Clin Nutr. 2019;38(3):1296–302. doi: 10.1016/j.clnu.2018.05.013 29914777

[4] Braun KV, Erler NS, Kiefte-de Jong JC, et al. Dietary intake of protein in early childhood is associated with growth trajectories between 1 and 9 years of age. J Nutr. 2016;146(11):2361–7. doi: 10.3945/jn.116.237164 27733529

[5] Voortman T, Braun KV, Kiefte-de Jong JC, et al. Protein intake in early childhood and body composition at the age of 6 years: the generation R study. Int J Obes (Lond). 2016;40(6):1018–1025. doi: 10.1038/ijo.2016.29 26975442

[6] Voortman T, van den Hooven EH, Tielemans MJ, et al. Protein intake in early childhood and cardiometabolic health at school age: the generation R study. Eur J Nutr. 2016;55(6):2117–2127. doi: 10.1007/s00394-015-1026-7 26329684 PMC5009172

[7] Durão C, Oliveira A, Santos AC, et al. Protein intake and dietary glycemic load of 4-year-olds and association with adiposity and serum insulin at 7 years of age: sex-nutrient and nutrient-nutrient interactions. Int J Obes (Lond). 2017;41(4):533–541. doi: 10.1038/ijo.2016.240 28028320

[8] Lu J, Gu Y, Liu H, et al. Daily branched-chain amino acid intake and risks of obesity and insulin resistance in children: a cross-sectional study. Obesity (Silver Spring). 2020;28(7):1310–1316. doi: 10.1002/oby.22834 32510827 PMC7311291

[9] Luque V, Closa-Monasterolo R, Escribano J, et al. Early programming by protein intake: the effect of protein on adiposity development and the growth and functionality of vital organs. Nutr Metab Insights. 2015;8(Suppl 1):49–56. doi: 10.4137/NMI.S29525 27013888 PMC4803318

[10] Lillycrop KA, Hoile SP, Grenfell L, et al. DNA methylation, ageing and the influence of early life nutrition. Proc Nutr Soc. 2014;73(3):413–421. doi: 10.1017/S0029665114000081 25027290

[11] Maddock J, Wulaningsih W, Fernandez JC, et al. Associations between body size, nutrition and socioeconomic position in early life and the epigenome: a systematic review. PLoS One. 2018;13(8):e0201672. doi: 10.1371/journal.pone.0201672 30096154 PMC6086410

[12] Joubert BR, den Dekker HT, Felix JF, et al. Maternal plasma folate impacts differential DNA methylation in an epigenome-wide meta-analysis of newborns. Nat Commun. 2016;7(1):10577. doi: 10.1038/ncomms10577 26861414 PMC4749955

[13] Briollais L, Rustand D, Allard C, et al. DNA methylation mediates the association between breastfeeding and early-life growth trajectories. Clin Epigenetics. 2021;13(1):231. doi: 10.1186/s13148-021-01209-z 34937578 PMC8697471

[14] Hartwig FP, Loret de Mola C, Davies NM, et al. Breastfeeding effects on DNA methylation in the offspring: a systematic literature review. PLoS One. 2017;12(3):e0173070. doi: 10.1371/journal.pone.0173070 28257446 PMC5336253

[15] Obermann-Borst SA, Eilers PH, Tobi EW, et al. Duration of breastfeeding and gender are associated with methylation of the LEPTIN gene in very young children. Pediatr Res. 2013;74(3):344–349. doi: 10.1038/pr.2013.95 23884163

[16] Hammad SS, Jones PJ. Dietary fatty acid composition modulates obesity and interacts with obesity-related genes. Lipids. 2017;52(10):803–22. doi: 10.1007/s11745-017-4291-9 28889206

[17] Voisin S, Almén MS, Moschonis G, et al. Dietary fat quality impacts genome-wide DNA methylation patterns in a cross-sectional study of Greek preadolescents. Eur J Hum Genet. 2015;23(5):654–62. doi: 10.1038/ejhg.2014.139 25074463 PMC4402618

[18] Perfilyev A, Dahlman I, Gillberg L, et al. Impact of polyunsaturated and saturated fat overfeeding on the DNA-methylation pattern in human adipose tissue: a randomized controlled trial. Am J Clin Nutr. 2017;105(4):991–1000. doi: 10.3945/ajcn.116.143164 28275132

[19] Waterland RA. Assessing the effects of high methionine intake on DNA methylation. J Nutr. 2006;136(6):1706S–10S. doi: 10.1093/jn/136.6.1706S 16702343

[20] Zheng J, Zhang L, Liu J, et al. Long-term effects of maternal low-protein diet and post-weaning high-fat feeding on glucose metabolism and hypothalamic POMC promoter methylation in offspring mice. Front Nutr. 2021;8:657848. doi: 10.3389/fnut.2021.657848 34485357 PMC8415226

[21] Altmann S, Murani E, Schwerin M, et al. Dietary protein restriction and excess of pregnant German Landrace sows induce changes in hepatic gene expression and promoter methylation of key metabolic genes in the offspring. J Nutr Biochem. 2013;24(2):484–95. doi: 10.1016/j.jnutbio.2012.01.011 22749136

[22] Altobelli G, Bogdarina IG, Stupka E, et al. Genome-wide methylation and gene expression changes in newborn rats following maternal protein restriction and reversal by folic acid. PLoS One. 2014;8(12):e82989. doi: 10.1371/journal.pone.0082989 PMC3877003 24391732

[23] Zaghlool SB, Kühnel B, Elhadad MA, et al. Epigenetics meets proteomics in an epigenome-wide association study with circulating blood plasma protein traits. Nat Commun. 2020;11(1):15. doi: 10.1038/s41467-019-13831-w 31900413 PMC6941977

[24] Ahsan M, Ek WE, Rask-Andersen M, et al. The relative contribution of DNA methylation and genetic variants on protein biomarkers for human diseases. PLoS Genet. 2017;13(9):e1007005. doi: 10.1371/journal.pgen.1007005 28915241 PMC5617224

[25] Koletzko B, von Kries R, Closa R, et al. Lower protein in infant formula is associated with lower weight up to age 2 y: a randomized clinical trial. Am J Clin Nutr. 2009;89(6):1836–45. doi: 10.3945/ajcn.2008.27091 19386747

[26] Kooijman MN, Kruithof CJ, van Duijn CM, et al. The generation R study: design and cohort update 2017. Eur J Epidemiol. 2016;31(12):1243–1264. doi: 10.1007/s10654-016-0224-9 28070760 PMC5233749

[27] Theurich MA, Zaragoza-Jordana M, Luque V, et al. Commercial complementary food use amongst European infants and children: results from the EU childhood obesity project. Eur J Nutr. 2020;59(4):1679–1692. doi: 10.1007/s00394-019-02023-3 31263982

[28] Schiess S, Grote V, Scaglioni S, et al. Introduction of complementary feeding in 5 European countries. J Pediatr Gastroenterol Nutr. 2010;50(1):92–8. doi: 10.1097/MPG.0b013e31819f1ddc 19543110

[29] Kiefte-de Jong JC, de Vries JH, Bleeker SE, et al. Socio-demographic and lifestyle determinants of ‘Western-like’ and ‘health conscious’ dietary patterns in toddlers. British Journal Of Nutrition. 2013;109(1):137–147. doi: 10.1017/S0007114512000682 22475342

[30] Lehne B, Drong AW, Loh M, et al. A coherent approach for analysis of the Illumina HumanMethylation450 BeadChip improves data quality and performance in epigenome-wide association studies. Genome Biol. 2015;16(1):37. doi: 10.1186/s13059-015-0600-x 25853392 PMC4365767

[31] Houseman EA, Accomando WP, Koestler DC, et al. DNA methylation arrays as surrogate measures of cell mixture distribution. BMC Bioinf. 2012;13(1):86. doi: 10.1186/1471-2105-13-86 PMC3532182 22568884

[32] Aryee MJ, Jaffe AE, Corrada-Bravo H, et al. Minfi: a flexible and comprehensive bioconductor package for the analysis of infinium DNA methylation microarrays. Bioinformatics. 2014;30(10):1363–9. doi: 10.1093/bioinformatics/btu049 24478339 PMC4016708

[33] R Core Team. R: a language and environment for statistical computing. Vienna, Austria: R Foundation for Statistical Computing; 2022. https://www.R-project.org/

[34] Reinius LE, Acevedo N, Joerink M, et al. Differential DNA methylation in purified human blood cells: implications for cell lineage and studies on disease susceptibility. PLoS One. 2012;7(7):e41361. doi: 10.1371/journal.pone.0041361 22848472 PMC3405143

[35] Venables WNR, D B. Modern applied statistics with S. Fourth ed. New York: Springer; 2002.

[36] Van der Most PJ, Küpers LK, Snieder H, et al. QCEWAS: automated quality control of results of epigenome-wide association studies. Bioinformatics. 2017;33(8):1243–1245. doi: 10.1093/bioinformatics/btw766 28119308

[37] Willer CJ, Li Y, Abecasis GR. METAL: fast and efficient meta-analysis of genomewide association scans. Bioinformatics. 2010;26(17):2190–1. doi: 10.1093/bioinformatics/btq340 20616382 PMC2922887

[38] Naeem H, Wong NC, Chatterton Z, et al. Reducing the risk of false discovery enabling identification of biologically significant genome-wide methylation status using the HumanMethylation450 array. BMC Genomics. 2014;15(1):51. doi: 10.1186/1471-2164-15-51 24447442 PMC3943510

[39] Chen YA, Lemire M, Choufani S, et al. Discovery of cross-reactive probes and polymorphic CpGs in the illumina infinium HumanMethylation450 microarray. Epigenetics. 2013;8(2):203–9. doi: 10.4161/epi.23470 23314698 PMC3592906

[40] Min JL, Hemani G, Hannon E, et al. Genomic and phenotypic insights from an atlas of genetic effects on DNA methylation. Nat Genet. 2021;53(9):1311–21. doi: 10.1038/s41588-021-00923-x 34493871 PMC7612069

[41] Benjamini Y, Hochberg Y. Controlling the false discovery rate: a practical and powerful approach to multiple testing. J Royal Stat Soc Ser B (Methodological). 1995;57(1):289–300. doi: 10.1111/j.2517-6161.1995.tb02031.x

[42] Battram T, Yousefi P, Crawford G, et al. The EWAS catalog: a database of epigenome-wide association studies. Wellcome Open Res. 2022;7:41. doi: 10.12688/wellcomeopenres.17598.2 35592546 PMC9096146

[43] Ruiz-Arenas C, Hernandez-Ferrer C, Vives-Usano M, et al. Identification of autosomal cis expression quantitative trait methylation (cis eQTMs) in children’s blood. Elife. 2022;11:11. doi: 10.7554/eLife.65310 PMC8933004 35302492

[44] Phipson B, Maksimovic J, Oshlack A. missMethyl: an R package for analyzing data from illumina’s HumanMethylation450 platform. Bioinformatics. 2016;32(2):286–8. doi: 10.1093/bioinformatics/btv560 26424855

[45] Nones K, Waddell N, Song S, et al. Genome-wide DNA methylation patterns in pancreatic ductal adenocarcinoma reveal epigenetic deregulation of SLIT-ROBO, ITGA2 and MET signaling. Int J Cancer. 2014;135(5):1110–8. doi: 10.1002/ijc.28765 24500968

[46] Wozniak MB, Le Calvez-Kelm F, Abedi-Ardekani B, et al. Integrative genome-wide gene expression profiling of clear cell renal cell carcinoma in Czech Republic and in the United States. PLoS One. 2013;8(3):e57886. doi: 10.1371/journal.pone.0057886 23526956 PMC3589490

[47] Ruiz-Arenas C, Hernandez-Ferrer C, Vives-Usano M, et al. Identification of autosomal cis expression quantitative trait methylation (cis eQTMs) in children’s blood. Elife. 2022;11:e65310. doi: 10.7554/eLife.65310 35302492 PMC8933004

[48] Bhagwandin C, Ashbeck EL, Whalen M, et al. The E3 ubiquitin ligase MARCH1 regulates glucose-tolerance and lipid storage in a sex-specific manner. PLoS One. 2018;13(10):e0204898. doi: 10.1371/journal.pone.0204898 30356278 PMC6200199

[49] Yang X-D, Xiang D-X, Yang Y-Y. Role of E3 ubiquitin ligases in insulin resistance. Diabetes Obesity Metab. 2016;18(8):747–54. doi: 10.1111/dom.12677 27097743

[50] Chen Y, Kassam I, Lau SH, et al. Impact of BMI and waist circumference on epigenome-wide DNA methylation and identification of epigenetic biomarkers in blood: an EWAS in multi-ethnic Asian individuals. Clin Epigenetics. 2021;13(1):195. doi: 10.1186/s13148-021-01162-x 34670603 PMC8527674

[51] Lee M, Kwon DY, Kim MS, et al. Genome-wide association study for the interaction between BMR and BMI in obese Korean women including overweight. Nutr Res Pract. 2016;10(1):115–24. doi: 10.4162/nrp.2016.10.1.115 26865924 PMC4742305

[52] Tachmazidou I, Süveges D, Min JL, et al. Whole-genome sequencing coupled to imputation discovers genetic signals for anthropometric traits. Am J Hum Genet. 2017;100(6):865–84. doi: 10.1016/j.ajhg.2017.04.014 28552196 PMC5473732

[53] Bonder MJ, Kasela S, Kals M, et al. Genetic and epigenetic regulation of gene expression in fetal and adult human livers. BMC Genomics. 2014;15(1):860. doi: 10.1186/1471-2164-15-860 25282492 PMC4287518

[54] Horvath S, Erhart W, Brosch M, et al. Obesity accelerates epigenetic aging of human liver. Proc Nat Acad Sci. 2014;111(43):15538–15543. doi: 10.1073/pnas.1412759111 25313081 PMC4217403

[55] Sjöstedt E, Zhong W, Fagerberg L, et al. An atlas of the protein-coding genes in the human, pig, and mouse brain. Science. 2020;367(6482):367(6482. doi: 10.1126/science.aay5947 32139519

[56] Sun J, Zhang X, Sun Y. C1orf109 promotes malignant phenotype of liver cancer via wnt signaling pathway in a CK2-dependent manner. J Mol Histol. 2023;54(2):135–45. doi: 10.1007/s10735-023-10117-w 36988773

[57] Stelzer G, Rosen N, Plaschkes I, et al. The GeneCards suite: from gene data mining to disease genome sequence analyses. Curr Protoc Bioinformatics. 2016;54(1):.1.30.1–.1.3. doi: 10.1002/cpbi.5 27322403

[58] Buniello A, MacArthur JAL, Cerezo M, et al. The NHGRI-EBI GWAS catalog of published genome-wide association studies, targeted arrays and summary statistics 2019. Nucleic Acids Res. 2019;47(D1):D1005–D12. doi: 10.1093/nar/gky1120 30445434 PMC6323933

[59] Totzauer M, Escribano J, Closa-Monasterolo R, et al. Different protein intake in the first year and its effects on adiposity rebound and obesity throughout childhood: 11 years follow-up of a randomized controlled trial. Pediatr Obes. 2022;17(12):e12961. doi: 10.1111/ijpo.12961 36355369

[60] Miousse IR, Pathak R, Garg S, et al. Short-term dietary methionine supplementation affects one-carbon metabolism and DNA methylation in the mouse gut and leads to altered microbiome profiles, barrier function, gene expression and histomorphology. Genes & Nutrition. 2017;12(1):22. doi: 10.1186/s12263-017-0576-0 28904640 PMC5588631

[61] Koletzko B, Demmelmair H, Grote V, et al. High protein intake in young children and increased weight gain and obesity risk1. Am J Clin Nutr. 2016;103(2):303–4. doi: 10.3945/ajcn.115.128009 26791192

[62] Breton CV, Landon R, Kahn LG, et al. Exploring the evidence for epigenetic regulation of environmental influences on child health across generations. Commun Biol. 2021;4(1):769. doi: 10.1038/s42003-021-02316-6 34158610 PMC8219763

[63] Nuru M, Muradashvili N, Kalani A, et al. High methionine, low folate and low vitamin B6/B12 (HM-LF-LV) diet causes neurodegeneration and subsequent short-term memory loss. Metab Brain Dis. 2018;33(6):1923–34. doi: 10.1007/s11011-018-0298-z 30094804 PMC6712979

[64] Zhang N. Role of methionine on epigenetic modification of DNA methylation and gene expression in animals. Anim Nutr. 2018;4(1):11–16. doi: 10.1016/j.aninu.2017.08.009 30167479 PMC6112339

[65] Assmann KE, Joslowski G, Buyken AE, et al. Prospective association of protein intake during puberty with body composition in young adulthood. Obesity. 2013;21(12):E782–E9. doi: 10.1002/oby.20516 23788493

[66] Damianidi L, Gruszfeld D, Verduci E, et al. Protein intakes and their nutritional sources during the first 2 years of life: secondary data evaluation from the European childhood obesity project. Eur J Clin Nutr. 2016;70(11):1291–7. doi: 10.1038/ejcn.2016.108 27329609

[67] Willett WC, Howe GR, Kushi LH. Adjustment for total energy intake in epidemiologic studies. Am J Clin Nutr. 1997;65(4 Suppl):1220S–1228S. discussion 9S-31S. doi: 10.1093/ajcn/65.4.1220S 9094926

[68] Joubert BR, Felix JF, Yousefi P, et al. DNA methylation in newborns and maternal smoking in pregnancy: genome-wide consortium meta-analysis. Am J Hum Genet. 2016;98(4):680–96. doi: 10.1016/j.ajhg.2016.02.019 27040690 PMC4833289

