# Supplementary material for: Animal and plant protein intake during infancy and childhood DNA methylation: a meta-analysis in the NutriPROGRAM consortium
Source: Epigenetics. 2024 Jan 10;19(1):2299045. doi: 10.1080/15592294.2023.2299045 (PMC10793674; doi:10.1080/15592294.2023.2299045)
Supplement: Supplementary.docx [file KEPI_A_2299045_SM6064.docx]

**Supplementary file**

**Tables:**

**Supplementary table 1.** Sex-stratified meta-analysis results for associations of infant animal protein intake (E%) and early childhood whole blood DNA methylation levels in boys at *P_FDR_* < 0.05.

|  |  |  |  |  |  |  |  |  |  |  | |  | |  | **Estimates in opposite sex (girls)** | | | | |
| --- | --- | --- | --- | --- | --- | --- | --- | --- | --- | --- | --- | --- | --- | --- | --- | --- | --- | --- | --- |
| **CpG** | **Effectᵃ** | **SEᵃ** | **P value** | **FDR** | **Directionᵇ** | **I²** | **Polymorphic** | **mQTLᶜ** | **Chr** | **Position** | **Relation to Island** | | **Nearest  Gene** | **Gene  Region** | **Effectᵃ** | **SEᵃ** | **P value** | **FDR** | **Directionᵇ** |
| cg04117356 | -0.00293 | 0.000495 | 3.09E-09 | 0.0012 | -- | 77.7 | Yes | Yes | 17 | 4406024 | S_Shelf | | SPNS2 | Body | 0.0005 | 0.0005 | 2.7E-01 | 0.91 | +- |
| cg25204440 | 0.00067 | 0.000115 | 6.66E-09 | 0.0013 | ++ | 82.9 | No | Yes | 1 | 209979598 | Island | | IRF6 | TSS200 | -0.0001 | 0.0002 | 3.8E-01 | 0.94 | -- |
| cg08714407 | -0.00059 | 0.000106 | 2.31E-08 | 0.0031 | -- | 89.5 | Yes | Yes | 2 | 24307071 | Island | | FAM228B | Body;1stExon | -0.0002 | 0.0001 | 2.3E-01 | 0.90 | -- |
| cg10409786 | -0.00093 | 0.000176 | 1.32E-07 | 0.0131 | +- | 87.8 | No | No | 20 | 35402300 | Island | | DSN1 | TSS200 | -0.0001 | 0.0002 | 6.0E-01 | 0.97 | -+ |
| cg26150300 | 0.00392 | 0.000751 | 1.79E-07 | 0.0142 | ++ | 72.7 | Yes | No | 6 | 134141433 | OpenSea | | LINC01312 | TSS1500 | 0.0003 | 0.0011 | 7.8E-01 | 0.99 | -+ |
| cg09175641 | -0.00092 | 0.000182 | 3.88E-07 | 0.0225 | -- | 0 | No | No | 13 | 53191471 | Island | | HNRNPA1L2 | TSS200 | -0.0003 | 0.0002 | 2.1E-01 | 0.89 | -- |
| cg09768257 | -0.00356 | 0.000703 | 4.25E-07 | 0.0225 | -- | 88.5 | No | Yes | 8 | 11628346 | S_Shore | | NEIL2 | 5'UTR | -0.0003 | 0.0008 | 6.8E-01 | 0.98 | -- |
| cg26664871 | 0.00063 | 0.000125 | 4.82E-07 | 0.0225 | -+ | 70.8 | No | No | 10 | 28032370 | Island | | MKX | 5'UTR | -0.0001 | 0.0001 | 5.0E-01 | 0.96 | -- |
| cg08789134 | 0.00064 | 0.000127 | 5.34E-07 | 0.0225 | +- | 80.6 | Yes | No | 4 | 6579201 | S_Shelf | | MAN2B2 | Body | -0.0001 | 0.0002 | 5.4E-01 | 0.96 | -+ |
| cg05620842 | -0.00049 | 0.000099 | 5.65E-07 | 0.0225 | -- | 0 | No | No | 19 | 34287343 | Island | | KCTD15 | TSS1500 | 0.0002 | 0.0001 | 1.3E-01 | 0.85 | +- |
| cg20171986 | 0.00196 | 0.000396 | 7.48E-07 | 0.0271 | ++ | 10.5 | No | Yes | 2 | 38830741 | Island | | HNRNPLL | TSS1500 | -0.0002 | 0.0006 | 7.7E-01 | 0.99 | -+ |
| cg07695269 | -0.00261 | 0.000530 | 8.63E-07 | 0.0287 | -- | 34.6 | No | No | 4 | 7394902 | OpenSea | | SORCS2 | Body | -0.0005 | 0.0006 | 3.9E-01 | 0.94 | -- |
| cg20471615 | -0.00149 | 0.000304 | 1.02E-06 | 0.0306 | -- | 46.7 | No | No | 9 | 139328837 | Island | | INPP5E | Body | 0.0002 | 0.0003 | 5.1E-01 | 0.96 | ++ |
| cg24899205 | -0.00040 | 0.000082 | 1.07E-06 | 0.0306 | -- | 55.7 | No | No | 22 | 19929184 | Island | | TXNRD2 | Body;TSS200 | 0.0001 | 0.0001 | 4.2E-01 | 0.95 | +- |
| cg19927065 | 0.00053 | 0.000110 | 1.35E-06 | 0.0357 | +- | 85.2 | No | No | 19 | 1122678 | Island | | SBNO2 | Body | -0.0003 | 0.0002 | 1.4E-01 | 0.86 | -- |
| cg16603374 | 0.00287 | 0.000596 | 1.48E-06 | 0.0367 | ++ | 84.3 | No | No | 1 | 6113068 | S_Shore | | KCNAB2 | Body | 0.0007 | 0.0005 | 1.7E-01 | 0.87 | ++ |

ᵃ Effect sizes and SE are presented as a percent change in DNA methylation per 1 E% increase in energy from animal protein intake.

ᵇ Studies are arranged from left to right as follows: CHOP then Generation R.

ᶜ methylation quantitative trait loci (mQTL) utilised from GoDMC database.

EWAS model was adjusted for total energy intake, age at blood collection, sex, maternal educational level, gestational age, pre-pregnancy BMI, maternal smoking during pregnancy, technical covariates, cell counts.

**Supplementary table 2.** Sex-stratified meta-analysis results for associations of infant animal protein intake (E%) and early childhood whole blood DNA methylation levels in girls at *P_FDR_* < 0.05.

|  |  |  |  |  |  |  |  |  |  |  |  |  |  | **Estimates in opposite sex (boys)** | | | | |
| --- | --- | --- | --- | --- | --- | --- | --- | --- | --- | --- | --- | --- | --- | --- | --- | --- | --- | --- |
| **CpG** | **Effectᵃ** | **SEᵃ** | **P value** | **FDR** | **Directionᵇ** | **I²** | **Polymorphic** | **mQTLᶜ** | **Chr** | **Position** | **Relation to Island** | **Nearest  Gene** | **Gene  Region** | **Effectᵃ** | **SEᵃ** | **P value** | **FDR** | **Directionᵇ** |
| cg01189915 | 0.00407 | 0.00065 | 4.01E-10 | 0.00016 | ++ | 54.8 | No | Yes | 7 | 1.28E+08 | N_Shore | HILPDA | TSS1500 | -0.001 | 0.001 | 1.2E-01 | 0.82 | -- |
| cg00322927 | -0.00785 | 0.00144 | 5.29E-08 | 0.00896 | -- | 82.9 | No | No | 11 | 1.33E+08 | OpenSea | OPCML | Body | -0.001 | 0.002 | 5.7E-01 | 0.96 | -+ |
| cg27530093 | 0.00214 | 0.00040 | 6.74E-08 | 0.00896 | ++ | 63.7 | No | No | 19 | 41815095 | N_Shore | CCDC97 | TSS1500 | 0.001 | 0.001 | 2.9E-01 | 0.91 | ++ |
| cg09486260 | -0.00063 | 0.00012 | 2.10E-07 | 0.01759 | -- | 61.2 | No | No | 19 | 10400648 | Island | ICAM5 | TSS200 | 0.000 | 0.000 | 5.4E-01 | 0.96 | -+ |
| cg19445690 | 0.00228 | 0.00044 | 2.37E-07 | 0.01759 | ++ | 0 | No | No | 2 | 74210890 | N_Shore | TET3 | - | 0.000 | 0.001 | 9.4E-01 | 1.00 | -+ |
| cg17890764 | 0.00277 | 0.00054 | 2.65E-07 | 0.01759 | ++ | 0 | No | Yes | 3 | 52864816 | OpenSea | MUSTN1 | TSS200 | -0.001 | 0.001 | 4.2E-01 | 0.94 | -+ |
| cg01513714 | 0.00134 | 0.00027 | 6.02E-07 | 0.03012 | ++ | 0 | No | No | 14 | 99738164 | N_Shore | BCL11B | TSS1500 | 0.000 | 0.000 | 5.1E-01 | 0.95 | -+ |
| cg01661350 | 0.00161 | 0.00032 | 6.04E-07 | 0.03012 | ++ | 0 | No | No | 13 | 70682098 | Island | KLHL1 | 1stExon;5'UTR;  Body | 0.001 | 0.000 | 9.9E-02 | 0.80 | ++ |
| cg25792714 | 0.00212 | 0.00043 | 9.73E-07 | 0.04310 | ++ | 0 | No | No | 6 | 21666554 | N_Shore | CASC15 | TSS200 | 0.001 | 0.001 | 3.3E-01 | 0.92 | ++ |

ᵃ Effect sizes and SE are presented as a percent change in DNA methylation per 1 E% increase in energy from animal protein intake.

ᵇ Studies are arranged from left to right as follows: CHOP then Generation R.

ᶜ methylation quantitative trait loci (mQTL) utilised from GoDMC database.

EWAS model was adjusted for total energy intake, age at blood collection, sex, maternal educational level, gestational age, pre-pregnancy BMI, maternal smoking during pregnancy, technical covariates, cell counts.

**Supplementary table 3.** Sex-stratified meta-analysis results for associations of infant animal protein intake (E%) and late childhood whole blood DNA methylation levels in boys at *P_FDR_* < 0.05.

| **CpG** | **Effectᵃ** | **SEᵃ** | **P value** | **FDR** | **Directionᵇ** | **I²** | **Polymorphic** | **mQTLᶜ** | **Chr** | **Position** | **Relation  to Island** | **Nearest Gene** | **Gene  Region** | **Estimates in opposite sex (girls)** | | | | |
| --- | --- | --- | --- | --- | --- | --- | --- | --- | --- | --- | --- | --- | --- | --- | --- | --- | --- | --- |
|  |  |  |  |  |  |  |  |  |  |  |  |  |  | **Effectᵃ** | **SEᵃ** | **P value** | **FDR** | **Directionᵇ** |
| cg15839394 | -0.00047 | 0.00009 | 1.36E-07 | 0.0321 | -- | 0 | No | No | 11 | 3013968 | Island | NAP1L4 | TSS1500 | -0.0001 | 0.0001 | 1.8E-01 | 0.87 | -- |
| cg12074025 | -0.00070 | 0.00013 | 2.31E-07 | 0.0321 | -- | 0 | No | No | 19 | 58238850 | Island | ZNF776 | 1stExon | 0.0002 | 0.0002 | 3.4E-01 | 0.93 | ++ |
| cg26730050 | -0.00328 | 0.00064 | 2.94E-07 | 0.0321 | -- | 0 | No | Yes | 13 | 1.13E+08 | OpenSea | SOX1 | - | -0.0010 | 0.0006 | 9.9E-02 | 0.81 | -- |
| cg01191254 | 0.00115 | 0.00023 | 3.71E-07 | 0.0321 | ++ | 6 | No | No | 7 | 2137963 | OpenSea | MAD1L1 | Body;Body; Body | -0.0005 | 0.0003 | 1.4E-01 | 0.84 | -- |
| cg11117266 | 0.00100 | 0.00020 | 4.39E-07 | 0.0321 | ++ | 40.2 | No | Yes | 17 | 43971461 | Island | MAPT-AS1 | TSS1500;Body | -0.0001 | 0.0003 | 6.6E-01 | 0.98 | -- |
| cg15538607 | 0.00228 | 0.00045 | 4.94E-07 | 0.0321 | ++ | 0 | No | Yes | 19 | 10416150 | N_Shelf | ZGLP1 | Body | 0.0012 | 0.0007 | 7.5E-02 | 0.79 | -+ |
| cg03304472 | -0.00136 | 0.00027 | 5.46E-07 | 0.0321 | -- | 70 | Yes | Yes | 5 | 94726091 | OpenSea | FAM81B | TSS1500 | -0.0008 | 0.0003 | 2.3E-02 | 0.67 | -- |
| cg02608124 | -0.00379 | 0.00077 | 8.97E-07 | 0.0461 | -- | 57.4 | Yes | Yes | 11 | 18193422 | OpenSea | MRGPRX4 | TSS1500 | 0.0004 | 0.0009 | 6.5E-01 | 0.97 | ++ |
| cg01391681 | -0.00117 | 0.00024 | 1.15E-06 | 0.0462 | -- | 0 | Yes | No | 7 | 1.42E+08 | OpenSea | PRSS37 | 1stExon;5'UTR | -0.0001 | 0.0003 | 6.3E-01 | 0.97 | -+ |
| cg18277754 | 0.00066 | 0.00014 | 1.16E-06 | 0.0462 | +- | 88.5 | No | No | 2 | 30144152 | Island | ALK | 5'UTR;1stExon | 0.0002 | 0.0002 | 3.8E-01 | 0.94 | ++ |
| cg21300373 | 0.00179 | 0.00037 | 1.24E-06 | 0.0462 | ++ | 3.9 | No | Yes | 4 | 1.65E+08 | Island | MARCHF1 | TSS200 | 0.0014 | 0.0004 | 7.6E-04 | 0.34 | ++ |

ᵃ Effect sizes and SE are presented as a percent change in DNA methylation per 1 E% increase in energy from animal protein intake.

ᵇ Studies are arranged from left to right as follows: CHOP then Generation R.

ᶜ methylation quantitative trait loci (mQTL) utilised from GoDMC database.

EWAS model was adjusted for total energy intake, age at blood collection, sex, maternal educational level, gestational age, pre-pregnancy BMI, maternal smoking during pregnancy, technical covariates, cell counts.

**Supplementary table 4.** Sex-stratified meta-analysis results for associations of infant animal protein intake (E%) and late childhood whole blood DNA methylation levels in girls at *P_FDR_* < 0.05.

| **CpG** | **Effectᵃ** | **SEᵃ** | **P value** | **FDR** | **Directionᵇ** | **I²** | **Polymorphic** | **mQTLᶜ** | **Chr** | **Position** | **Relation to  Island** | **Nearest  Gene** | **Gene  Region** | **Estimates in opposite sex (boys)** | | | | |
| --- | --- | --- | --- | --- | --- | --- | --- | --- | --- | --- | --- | --- | --- | --- | --- | --- | --- | --- |
|  |  |  |  |  |  |  |  |  |  |  |  |  |  | **Effectᵃ** | **SEᵃ** | **P value** | **FDR** | **Directionᵇ** |
| cg09022552 | -0.00217 | 0.00036 | 1.79E-09 | 0.001 | -- | 0 | No | No | 16 | 28962119 | Island | NFATC2IP | TSS200 | -0.0001 | 0.0004 | 8.5E-01 | 0.99 | -+ |

ᵃ Effect sizes and SE are presented as a percent change in DNA methylation per 1 E% increase in energy from animal protein intake.

ᵇ Studies are arranged from left to right as follows: CHOP then Generation R.

ᶜ methylation quantitative trait loci (mQTL) utilised from GoDMC database.

EWAS model was adjusted for total energy intake, age at blood collection, sex, maternal educational level, gestational age, pre-pregnancy BMI, maternal smoking during pregnancy, technical covariates, cell counts.

**Supplementary table 5.** Sex-stratified meta-analysis results for associations of infant plant protein intake (E%) and early childhood whole blood DNA methylation levels in boys at *P_FDR_* < 0.05.

| **CpG** | **Effectᵃ** | **SEᵃ** | **P value** | **FDR** | **Directionᵇ** | **I²** | **Polymorphic** | **mQTLᶜ** | **Chr** | **Position** | **Relation to  Island** | **Nearest  Gene** | **Gene  Region** | **Estimates in opposite sex (girls)** | | | | |
| --- | --- | --- | --- | --- | --- | --- | --- | --- | --- | --- | --- | --- | --- | --- | --- | --- | --- | --- |
|  |  |  |  |  |  |  |  |  |  |  |  |  |  | **Effectᵃ** | **SEᵃ** | **P value** | **FDR** | **Directionᵇ** |
| cg09508434 | 0.0036 | 0.0007 | 9.21E-08 | 0.037 | ++ | 0 | Yes | Yes | 5 | 1.11E+08 | OpenSea | STARD4-AS1 | - | 0.0003 | 0.0009 | 7.7E-01 | 0.992 | -+ |

ᵃ Effect sizes and SE are presented as a percent change in DNA methylation per 1 E% increase in energy from animal protein intake.

ᵇ Studies are arranged from left to right as follows: CHOP then Generation R.

ᶜ methylation quantitative trait loci (mQTL) utilised from GoDMC database.

EWAS model was adjusted for total energy intake, age at blood collection, sex, maternal educational level, gestational age, pre-pregnancy BMI, maternal smoking during pregnancy, technical covariates, cell counts.

**Supplementary table 6.** Sex-stratified meta-analysis results for associations of infant plant protein intake (E%) and early childhood whole blood DNA methylation levels in girls at *P_FDR_* < 0.05.

| **CpG** | **Effectᵃ** | **SEᵃ** | **P value** | **FDR** | **Directionᵇ** | **I²** | **Polymorphic** | **mQTLᶜ** | **Chr** | **Position** | **Relation to  Island** | **Nearest Gene** | **Gene  Region** | **Estimates in opposite sex (boys)** | | | | |
| --- | --- | --- | --- | --- | --- | --- | --- | --- | --- | --- | --- | --- | --- | --- | --- | --- | --- | --- |
|  |  |  |  |  |  |  |  |  |  |  |  |  |  | **Effectᵃ** | **SEᵃ** | **P value** | **FDR** | **Directionᵇ** |
| cg16732341 | -0.0055 | 0.0009 | 1.42E-09 | 0.0006 | -- | 0 | Yes | No | 4 | 112130812 | OpenSea | PITX2 | - | -0.001 | 0.001 | 5.5E-01 | 0.98 | -- |
| cg13847137 | -0.0031 | 0.0005 | 1.15E-08 | 0.0023 | -- | 55.3 | Yes | No | 4 | 6380703 | S_Shore | PPP2R2C | Body;Body | 0.001 | 0.001 | 3.9E-01 | 0.96 | ++ |
| cg23258065 | 0.0013 | 0.0002 | 2.05E-08 | 0.0027 | ++ | 75.5 | No | No | 20 | 442606 | Island | TBC1D20 | Body | 0.000 | 0.000 | 4.1E-01 | 0.96 | -- |

ᵃ Effect sizes and SE are presented as a percent change in DNA methylation per 1 E% increase in energy from animal protein intake.

ᵇ Studies are arranged from left to right as follows: CHOP then Generation R.

ᶜ methylation quantitative trait loci (mQTL) utilised from GoDMC database.

EWAS model was adjusted for total energy intake, age at blood collection, sex, maternal educational level, gestational age, pre-pregnancy BMI, maternal smoking during pregnancy, technical covariates, cell counts.

**Supplementary table 7.** Sex-stratified meta-analysis results for associations of infant plant protein intake (E%) and late childhood whole blood DNA methylation levels in boys at *P_FDR_* < 0.05.

| **CpG** | **Effectᵃ** | **SEᵃ** | **P value** | **FDR** | **Directionᵇ** | **I²** | **Polymorphic** | **mQTLᶜ** | **Chr** | **Position** | **Relation  to Island** | **Nearest Gene** | **Gene  Region** | **Estimates in opposite sex (girls)** | | | | |
| --- | --- | --- | --- | --- | --- | --- | --- | --- | --- | --- | --- | --- | --- | --- | --- | --- | --- | --- |
|  |  |  |  |  |  |  |  |  |  |  |  |  |  | **Effectᵃ** | **SEᵃ** | **P value** | **FDR** | **Directionᵇ** |
| cg17343184 | 0.01103 | 0.00168 | 5.32E-11 | 0.00002 | ++ | 0 | No | No | 2 | 189834269 | OpenSea | COL3A1 | - | 0.0004 | 0.0025 | 8.7E-01 | 0.99 | +- |
| cg01442068 | -0.00248 | 0.00045 | 3.54E-08 | 0.00729 | -- | 0 | No | Yes | 17 | 53641714 | S_Shelf | MMD | - | 0.0003 | 0.0007 | 6.5E-01 | 0.98 | +- |
| cg19682639 | -0.00541 | 0.00103 | 1.69E-07 | 0.02311 | -- | 0 | No | Yes | 1 | 1391102 | S_Shore | ATAD3C | Body | 0.0016 | 0.0012 | 1.6E-01 | 0.87 | ++ |
| cg10438560 | 0.00323 | 0.00065 | 6.41E-07 | 0.04865 | ++ | 77 | No | No | 1 | 53642234 | OpenSea | CPT2 | - | 0.0008 | 0.0007 | 2.6E-01 | 0.91 | ++ |
| cg05994794 | 0.00302 | 0.00061 | 7.03E-07 | 0.04865 | ++ | 67 | No | No | 4 | 41752248 | N_Shore | PHOX2B | TSS1500 | -0.0014 | 0.0006 | 1.8E-02 | 0.65 | -- |
| cg22892036 | 0.00328 | 0.00066 | 7.71E-07 | 0.04865 | ++ | 66 | No | Yes | 7 | 28189139 | OpenSea | JAZF1 | Body | 0.0009 | 0.0010 | 3.8E-01 | 0.94 | -+ |
| cg15015119 | 0.00475 | 0.00097 | 9.58E-07 | 0.04865 | ++ | 70 | No | Yes | 5 | 41587896 | OpenSea | PLCXD3 | - | -0.0021 | 0.0012 | 9.2E-02 | 0.81 | -- |
| cg14879574 | 0.00993 | 0.00203 | 1.03E-06 | 0.04865 | ++ | 0 | No | No | 2 | 200136686 | OpenSea | SATB2 | 3'UTR | -0.0038 | 0.0028 | 1.6E-01 | 0.87 | +- |
| cg22221575 | -0.00747 | 0.00153 | 1.06E-06 | 0.04865 | -- | 53 | No | Yes | 3 | 195965369 | OpenSea | PCYT1A | 3'UTR | -0.0010 | 0.0020 | 6.2E-01 | 0.97 | -- |

ᵃ Effect sizes and SE are presented as a percent change in DNA methylation per 1 E% increase in energy from animal protein intake.

ᵇ Studies are arranged from left to right as follows: CHOP then Generation R.

ᶜ methylation quantitative trait loci (mQTL) utilised from GoDMC database.

EWAS model was adjusted for total energy intake, age at blood collection, sex, maternal educational level, gestational age, pre-pregnancy BMI, maternal smoking during pregnancy, technical covariates, cell counts.

**Supplementary table 8.** Sex-stratified meta-analysis results for associations of infant plant protein intake (E%) and late childhood whole blood DNA methylation levels in girls at *P_FDR_* < 0.05.

| **CpG** | **Effectᵃ** | **SEᵃ** | **P value** | **FDR** | **Directionᵇ** | **I²** | **Polymorphic** | **mQTLᶜ** | **Chr** | **Position** | **Relation  to Island** | **Nearest Gene** | **Gene  Region** | **Estimates in opposite sex (boys)** | | | | |
| --- | --- | --- | --- | --- | --- | --- | --- | --- | --- | --- | --- | --- | --- | --- | --- | --- | --- | --- |
|  |  |  |  |  |  |  |  |  |  |  |  |  |  | **Effectᵃ** | **SEᵃ** | **P value** | **FDR** | **Directionᵇ** |
| cg27558485 | 0.00723 | 0.00126 | 9.33E-09 | 0.004 | ++ | 0 | No | Yes | 3 | 1.96E+08 | OpenSea | SLC51A | TSS1500 | 0.001 | 0.002 | 7.8E-01 | 0.98 | -+ |

ᵃ Effect sizes and SE are presented as a percent change in DNA methylation per 1 E% increase in energy from animal protein intake.

ᵇ Studies are arranged from left to right as follows: CHOP then Generation R.

ᶜ methylation quantitative trait loci (mQTL) utilised from GoDMC database.

EWAS model was adjusted for total energy intake, age at blood collection, sex, maternal educational level, gestational age, pre-pregnancy BMI, maternal smoking during pregnancy, technical covariates, cell counts.

**Supplementary table 9.**  Longitudinal look-up of CpGs significant at either timepoints with animal and plant protein intake at FDR < 0.05.

|  | **Animal protein** | | | | | | | | | |
| --- | --- | --- | --- | --- | --- | --- | --- | --- | --- | --- |
|  | **Early childhood** | | | | | **Late childhood** | | | | |
| **CpG** | **Effectᵃ** | **SEᵃ** | **P value** | **FDR** | **I²** | **Effectᵃ** | **SEᵃ** | **P Value** | **FDR** | **I²** |
| cg21300373 | 0.0006 | 0.0004 | 0.13 | 0.84 | 0 | 0.16 | 0.03 | 4.27E-08 | 0.018 | 0 |
| cg10633363 | 0.0001 | 0.0002 | 0.70 | 0.97 | 21 | -0.05 | 0.01 | 1.09E-07 | 0.022 | 50.6 |
|  | **Plant protein** | | | | | | | | | |
|  | **Early childhood** | | | | | **Late childhood** | | | | |
| **CpG** | **Effectᵃ** | **SEᵃ** | **P value** | **FDR** | **I²** | **Effectᵃ** | **SEᵃ** | **P Value** | **FDR** | **I²** |
| cg15407373 | -0.003 | 0.0005 | 2.13E-07 | 0.047 | 95.6 | -0.0001 | 0.0006 | 0.80 | 1.00 | 0 |
| cg25973293 | -0.003 | 0.0006 | 2.26E-07 | 0.047 | 5.2 | -0.0009 | 0.0005 | 0.09 | 0.96 | 0 |

ᵃ Effect sizes and SE are presented as a percent change in DNA methylation per 1 E% increase in energy from animal protein intake.

**Figures:**


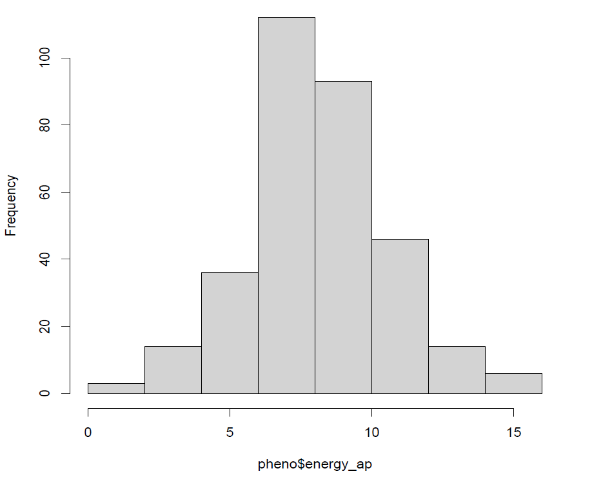


**B. Generation R - Early childhood**


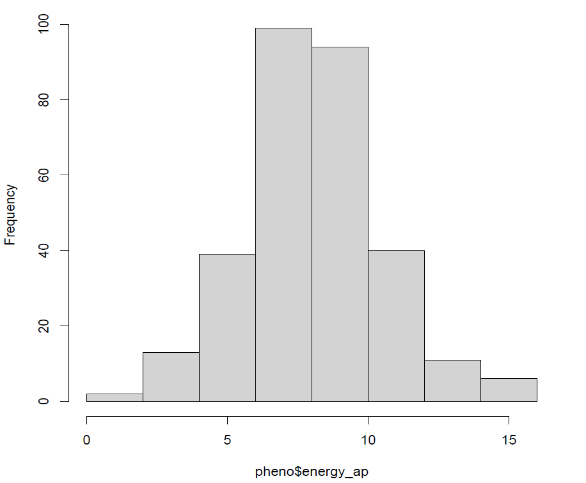


**D. Generation R – Late childhood**


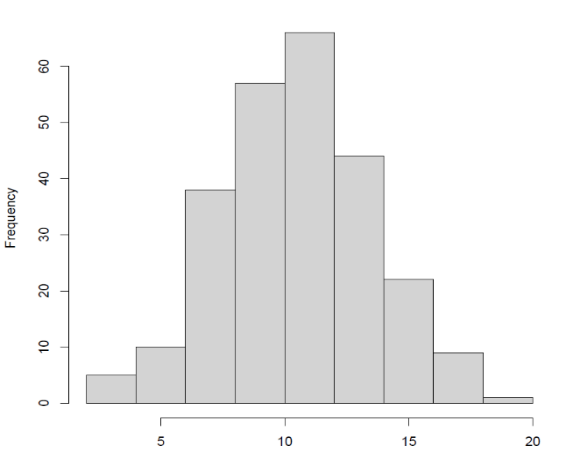


**A. CHOP - Early childhood**


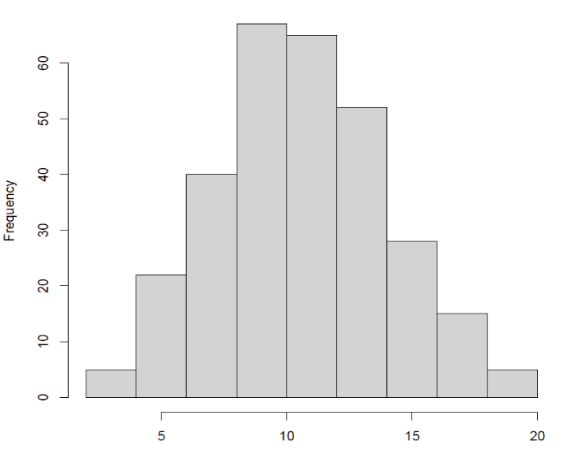


**C. CHOP - Late childhood**

**Supplementary figure 1.** Study-specific distribution of infant animal protein intake (E%).

**B. Generation R - Early childhood**

**D. Generation R – Late childhood**

**A. CHOP - Early childhood**

**C. CHOP - Late childhood**


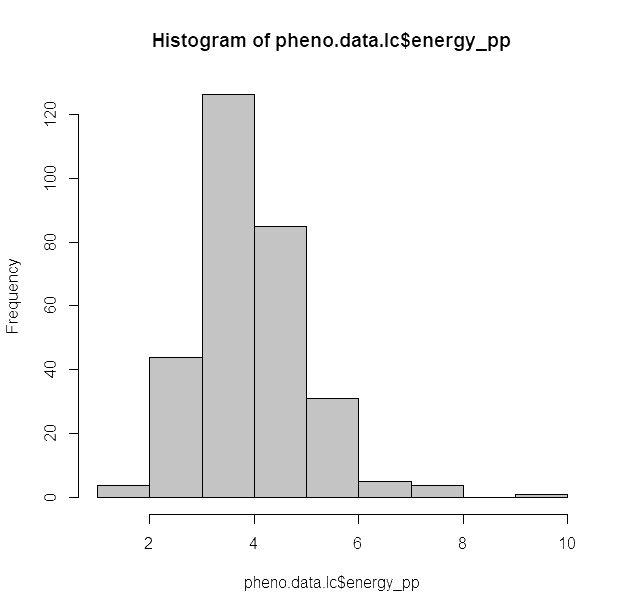

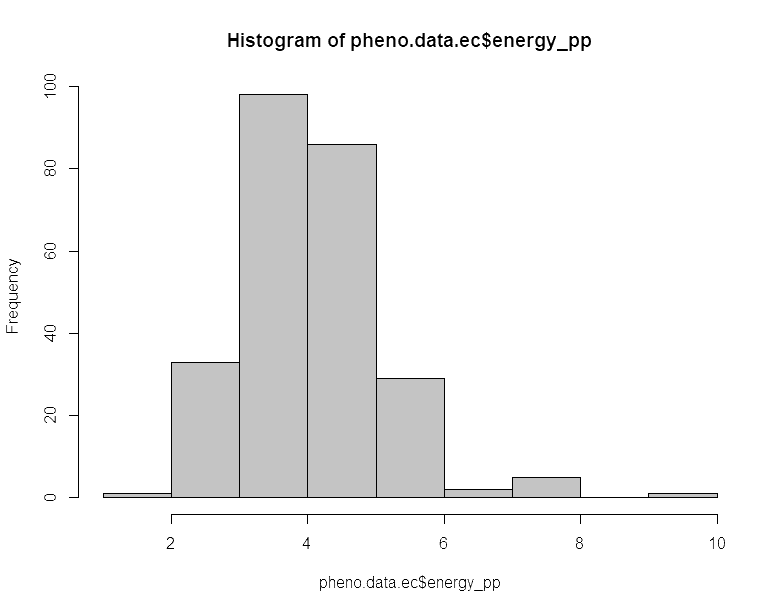

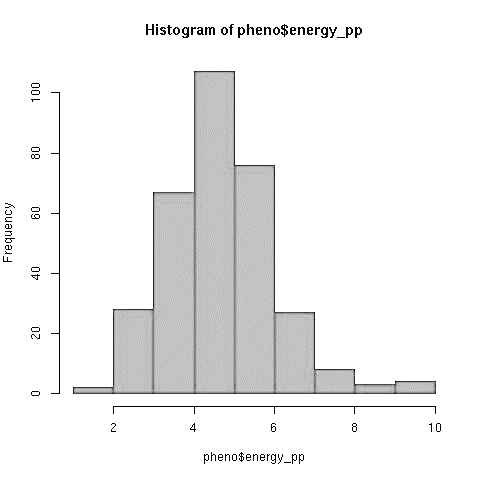

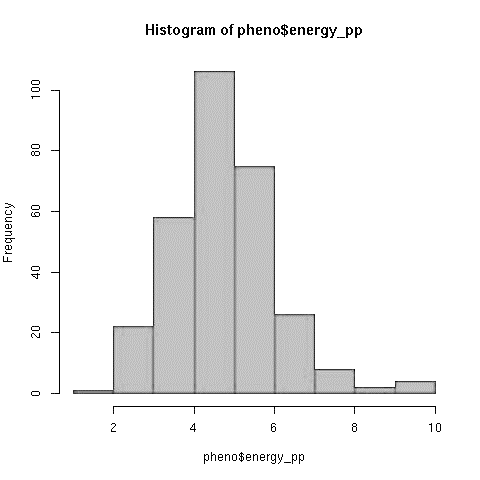


**Supplementary figure 2.** Study-specific distribution of infant plant protein intake (E%).


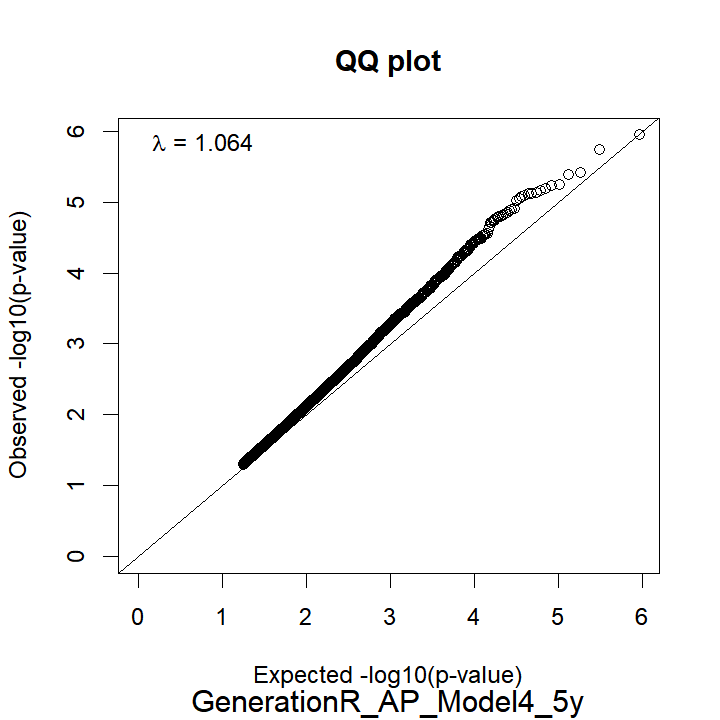

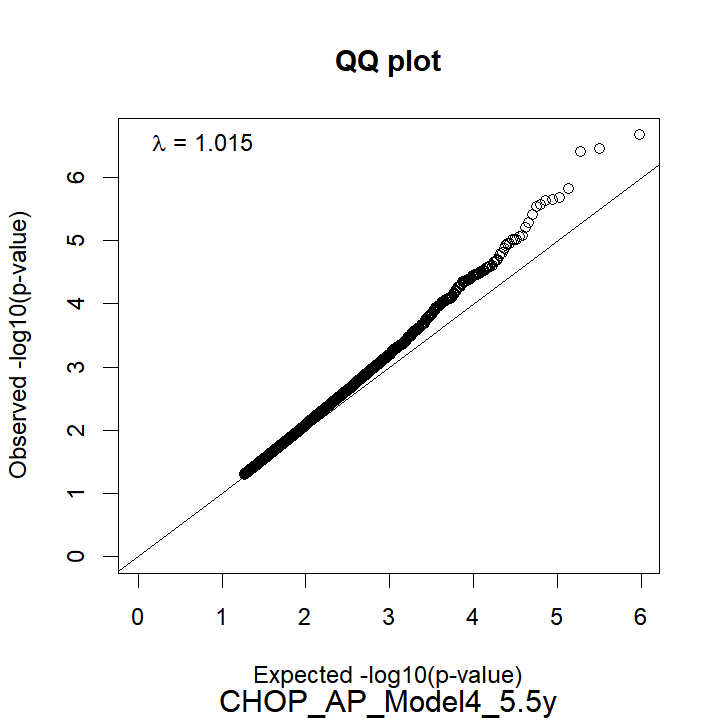


**A. CHOP Early childhood**

**C. Generation R Early childhood**


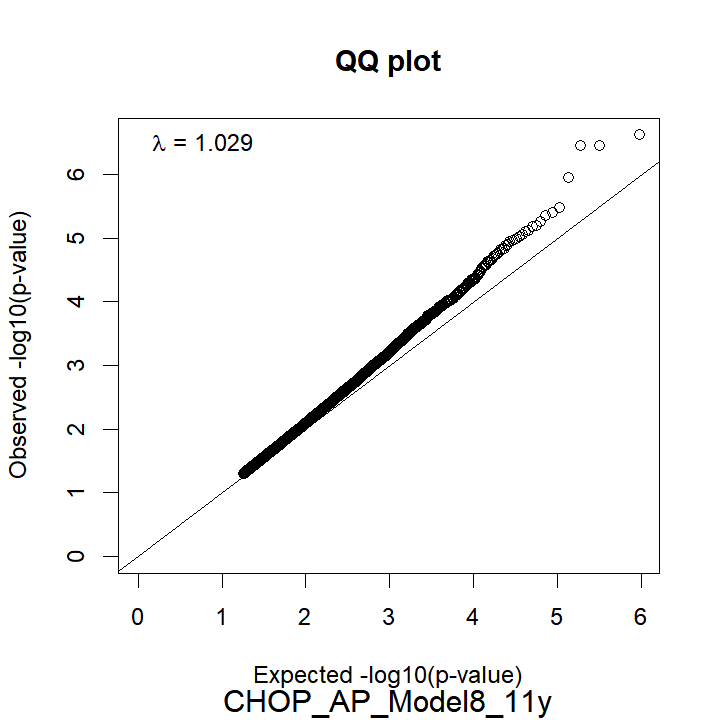

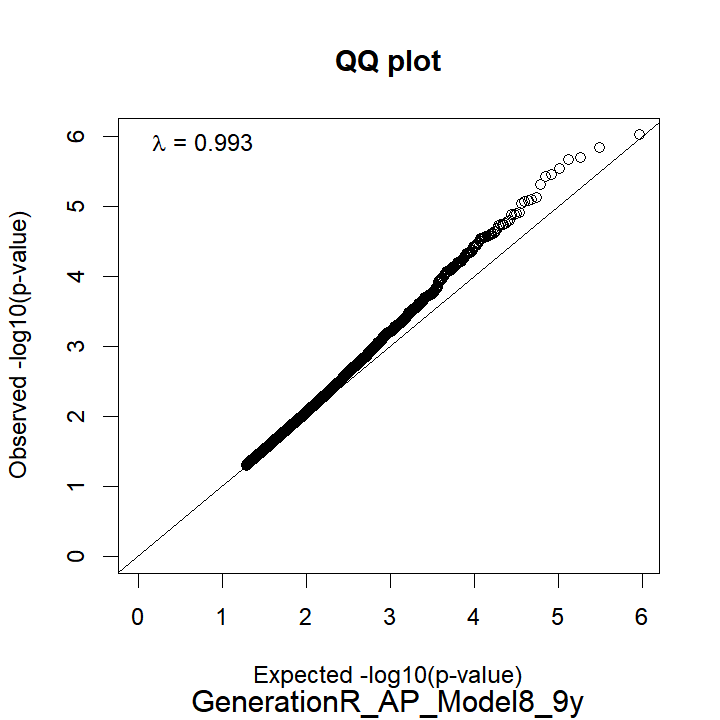


**B. CHOP Late childhood**

**D. Generation R Late childhood**

**Supplementary figure 3.** Q-Q plots and lambdas for fully adjusted EWAS models of E% intake from animal protein sources at1^st^ year of age in both study with whole blood DNA methylation at early and late childhood.

**
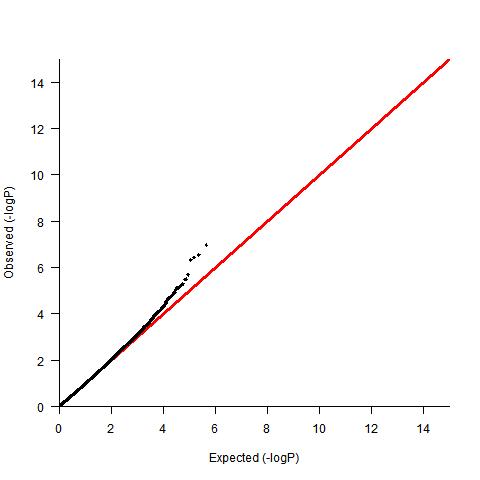
**
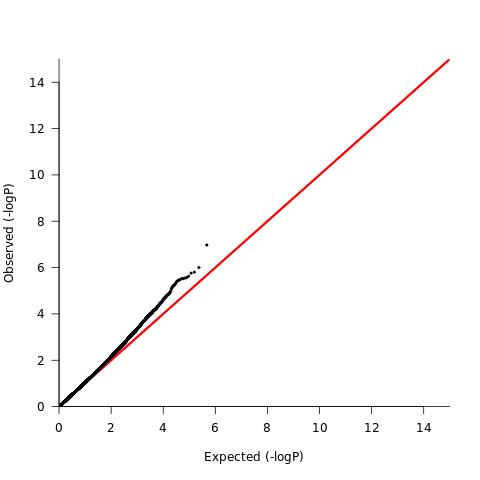

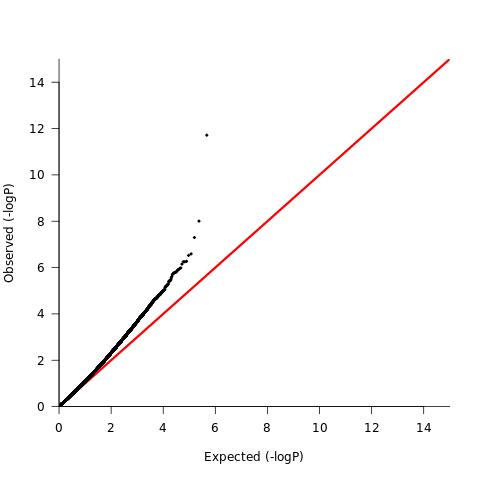

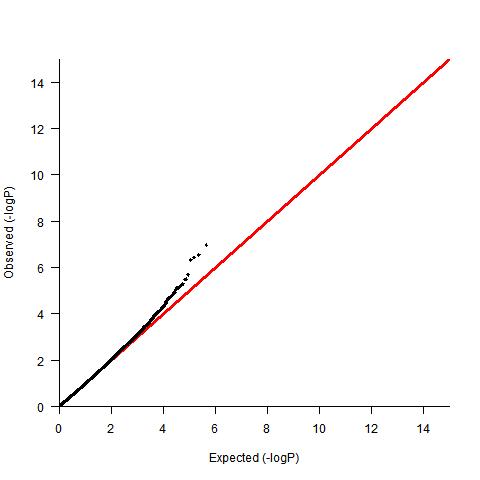


λ = 0.907

**A. CHOP Early childhood**

**C. Generation R Early childhood**

**B. CHOP Late childhood**

**D. Generation R Late childhood**

λ = 0.943

λ =1.03

λ =1.08

**Supplementary figure 4.** Q-Q plots and lambdas for fully adjusted EWAS models of E% intake from plant protein sources at1^st^ year of age in both study with whole blood DNA methylation at early and late childhood.


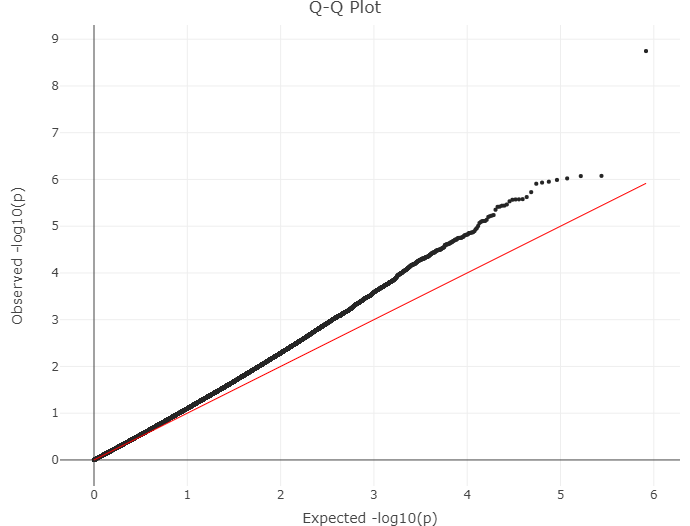

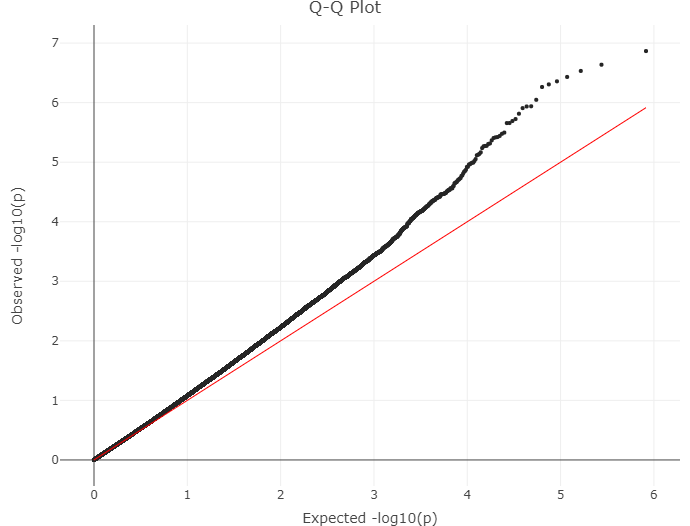

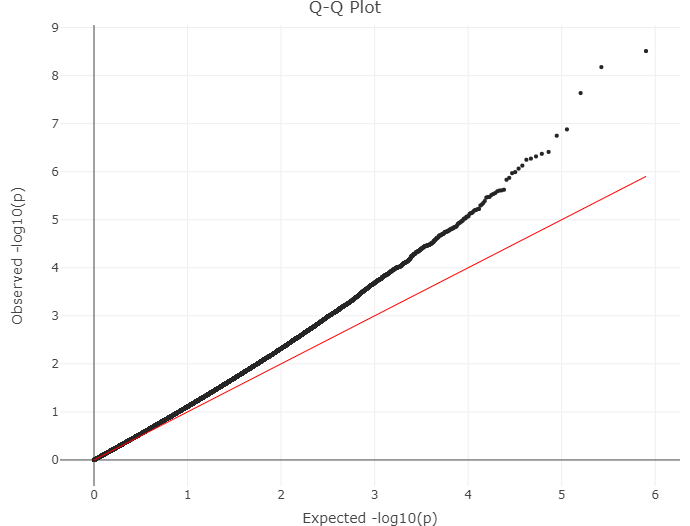

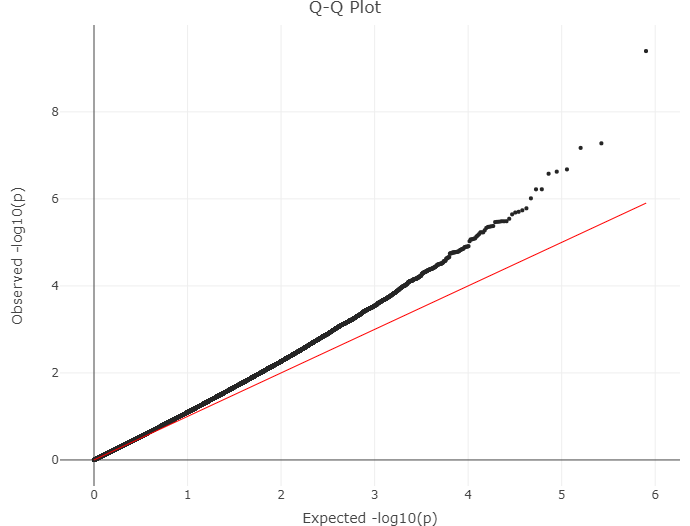


λ =1.12

λ =1.10

**(c) QQ plot late childhood – Animal Protein males**

**(d) QQ plot late childhood – Animal Protein females**

λ =1.11

**Supplementary figure 5**. Q-Q plots and lambda for sex-stratified associations between infant animal protein intake (E%) and early and late childhood DNA methylation.

λ =1.08

**(b) QQ plot early childhood – Animal Protein females**

**(a) QQ plot early childhood** **– Animal Protein males**


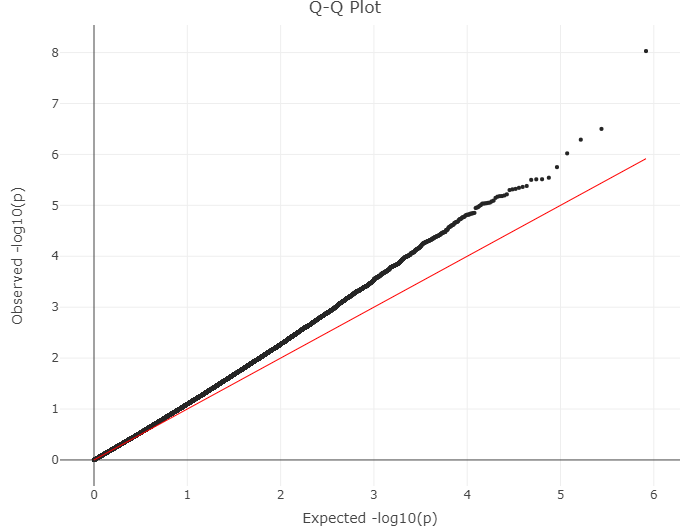
*
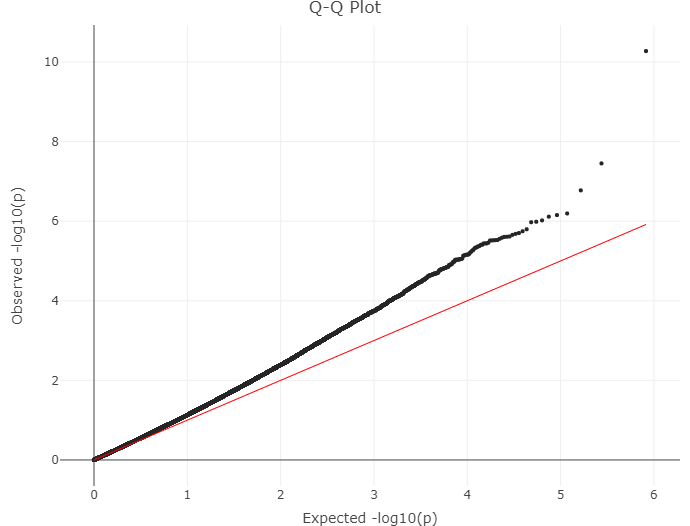
*
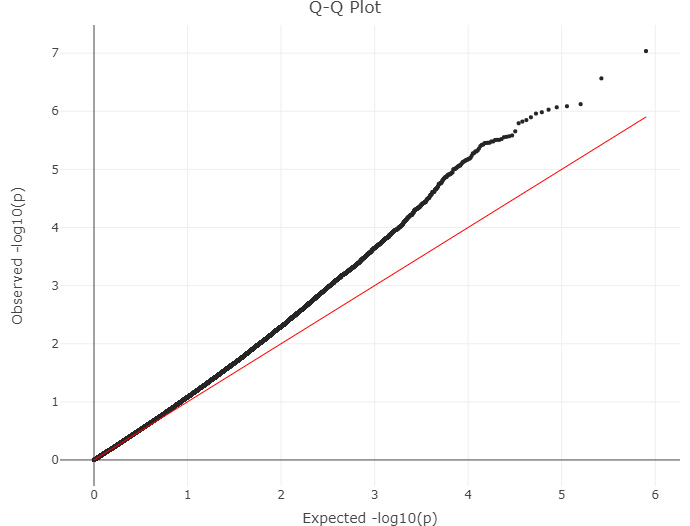

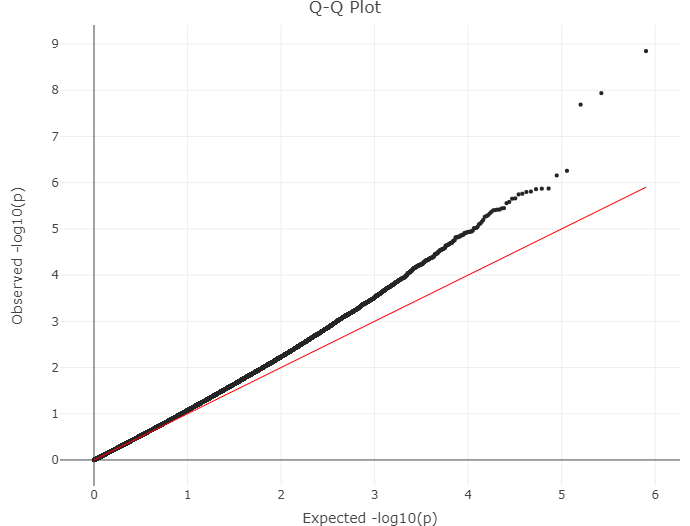


λ =1.11

λ =1.14

λ =1.07

λ =1.07

**(c) QQ plot late childhood – Plant Protein males**

**(d) QQ plot late childhood – Plant Protein females**

**Supplementary figure 6**. Q-Q plots and lambda for sex-stratified associations between infant plant protein intake (E%) and early and late childhood DNA methylation.

**(b) QQ plot early childhood – Plant Protein females**

**(a) QQ plot early childhood – Plant Protein males**


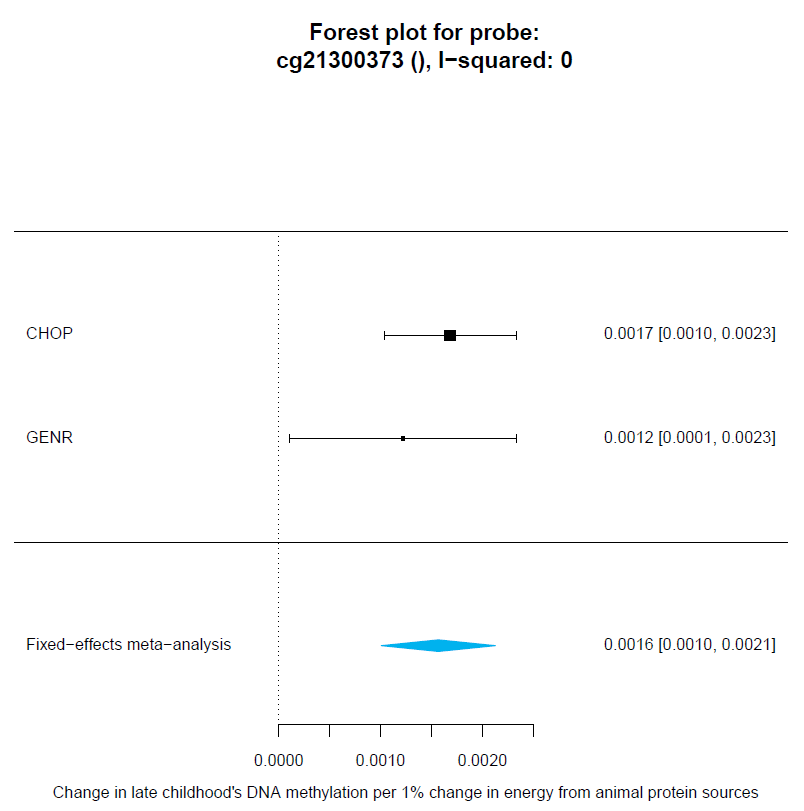


**Supplementary figure 7.** Forest plot for cg21300373 significant with animal protein intake


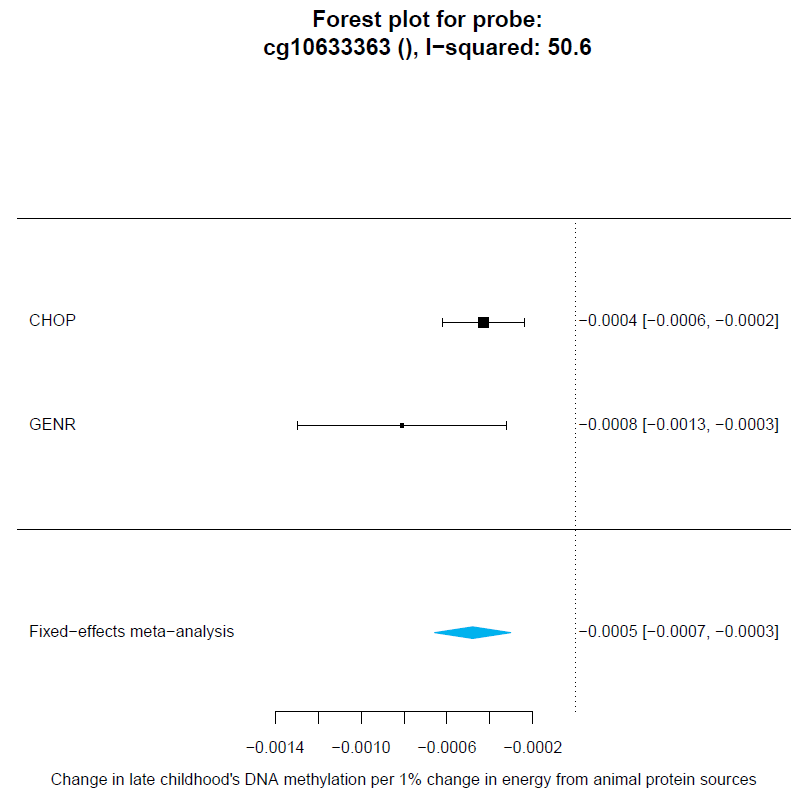


**Supplementary figure 8.** Forest plot for cg10633363 significant with animal protein intake


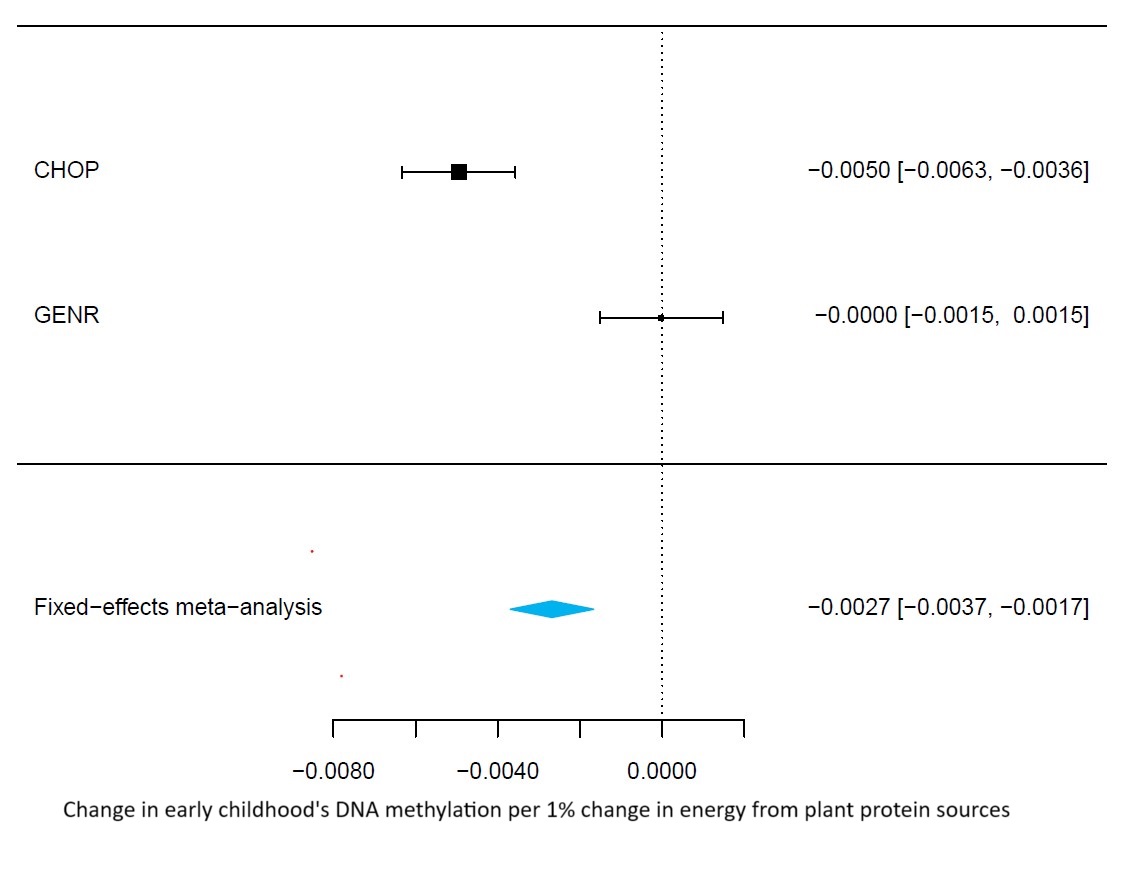


**Supplementary figure 9.** Forest plot for cg15407373 significant with plant protein intake


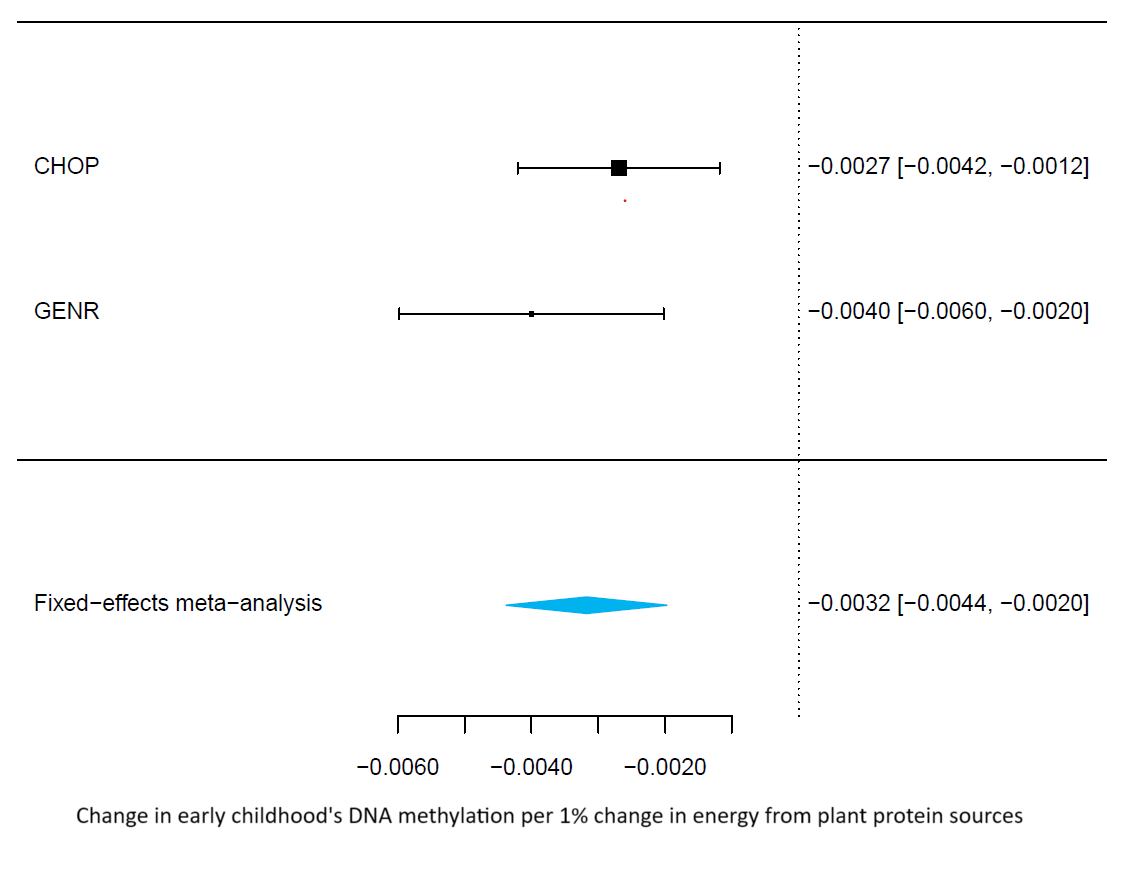


**Supplementary figure 10.** Forest plot for cg25973293 significant with plant protein intake

**Supplementary Figure 11.** Hartigan’s dip test for unimodality for cg21300373 in both cohorts at late childhood. A: CHOP, D = 0.015, p = 0.97. B: Generation R, D = 0.014, p = 0.96.

**A. CHOP**

**B. Generation R**

**A. CHOP**

**B. Generation R**

**Supplementary Figure 12.** Hartigan’s dip test for unimodality for cg10633363 in both cohorts at late childhood. A. CHOP: D = 0.011, p = 0.99. B. Generation R: D = 0.010, p = 0.98.

**A. CHOP**

**B. Generation R**


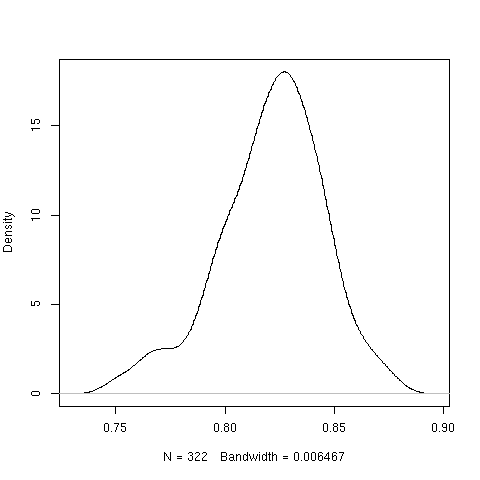
**
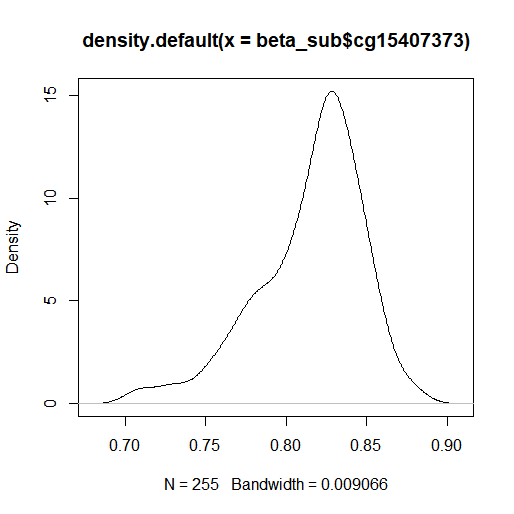
**

**Supplementary Figure 13.** Hartigan’s dip test for unimodality for cg15407373 in both cohorts at early childhood. A: CHOP, D = 0.015, p = 0.98. B: Generation R, D = 0.011, p = 0.99.


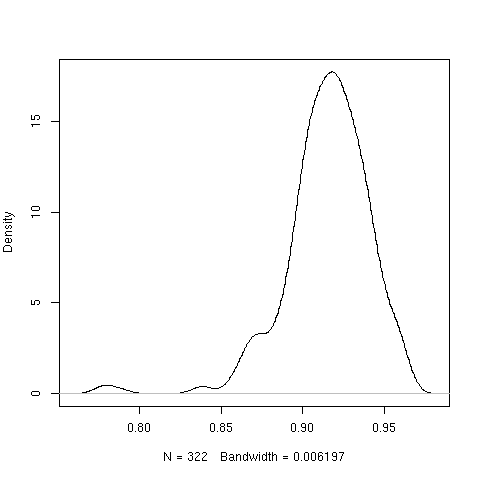
**
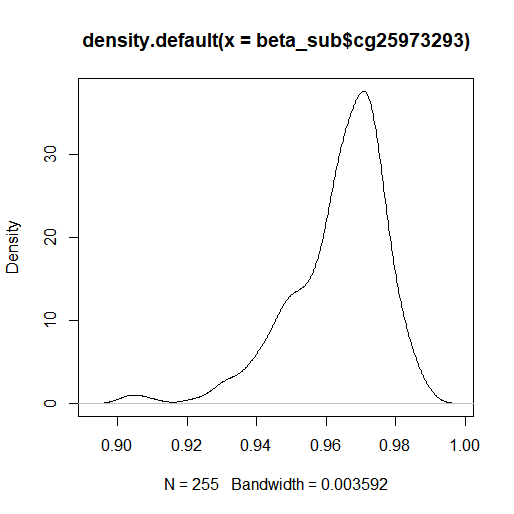
**

**A. CHOP**

**B. Generation R**

**Supplementary Figure 14.** Hartigan’s dip test for unimodality for cg25973293 in both cohorts at early childhood. A: CHOP, D = 0.015, p = 0.96. B: Generation R, D = 0.015, p = 0.95.
